# CTCF modulates allele-specific sub-TAD organization and imprinted gene activity at the mouse *Dlk1-Dio3* and *Igf2-H19* domains

**DOI:** 10.1186/s13059-019-1896-8

**Published:** 2019-12-12

**Authors:** David Llères, Benoît Moindrot, Rakesh Pathak, Vincent Piras, Mélody Matelot, Benoît Pignard, Alice Marchand, Mallory Poncelet, Aurélien Perrin, Virgile Tellier, Robert Feil, Daan Noordermeer

**Affiliations:** 1 Institute of Molecular Genetics of Montpellier (IGMM), University of Montpellier, CNRS, Montpellier, France; 2 Institute for Integrative Biology of the Cell (I2BC), CEA, CNRS, University Paris-sud and University Paris-Saclay, Gif-sur-Yvette, France

**Keywords:** Genomic imprinting, 3D genome organization, *Dlk1*, *Meg3*, *Igf2*, CTCF, Topologically associating domains, TADs, DNA methylation, Development

## Abstract

**Background:**

Genomic imprinting is essential for mammalian development and provides a unique paradigm to explore intra-cellular differences in chromatin configuration. So far, the detailed allele-specific chromatin organization of imprinted gene domains has mostly been lacking. Here, we explored the chromatin structure of the two conserved imprinted domains controlled by paternal DNA methylation imprints—the *Igf2-H19* and *Dlk1-Dio3* domains—and assessed the involvement of the insulator protein CTCF in mouse cells.

**Results:**

Both imprinted domains are located within overarching topologically associating domains (TADs) that are similar on both parental chromosomes. At each domain, a single differentially methylated region is bound by CTCF on the maternal chromosome only, in addition to multiple instances of bi-allelic CTCF binding. Combinations of allelic 4C-seq and DNA-FISH revealed that bi-allelic CTCF binding alone, on the paternal chromosome, correlates with a first level of sub-TAD structure. On the maternal chromosome, additional CTCF binding at the differentially methylated region adds a further layer of sub-TAD organization, which essentially hijacks the existing paternal-specific sub-TAD organization. Perturbation of maternal-specific CTCF binding site at the *Dlk1-Dio3* locus, using genome editing, results in perturbed sub-TAD organization and bi-allelic *Dlk1* activation during differentiation.

**Conclusions:**

Maternal allele-specific CTCF binding at the imprinted *Igf2-H19* and the *Dlk1-Dio3* domains adds an additional layer of sub-TAD organization, on top of an existing three-dimensional configuration and prior to imprinted activation of protein-coding genes. We speculate that this allele-specific sub-TAD organization provides an instructive or permissive context for imprinted gene activation during development.

## Background

Genomic imprinting is an epigenetic mechanism through which parental origin dictates mono-allelic expression of about 200 mammalian genes [1]. Imprinting is essential for embryonic development, with diverse disease syndromes in humans attributed to loss of parental specificity [2]. The majority of imprinted genes are clustered in about 20 chromosomal domains, where “Imprinting Control Regions” (ICRs) dictate allele-specific gene activity. All ICRs are differentially methylated regions (DMRs) that carry germline-acquired allelic DNA methylation imprints [3].

Most ICRs are methylated on the maternally inherited chromosome, where they overlap promoters that are expressed only from the paternal chromosome. In contrast, only at two evolutionary conserved domains (*Igf2-H19* and *Dlk1-Dio3*), the ICR is methylated on the paternal allele. Uniquely, these “paternal ICRs” do not overlap promoters but are linked to allele-specific binding of the CTCF insulator protein on the maternal chromosome, either directly to the ICR itself (“*H19* DMR” in the *Igf2-H19* domain), or to a secondary DMR whose allelic methylation during pre-implantation development requires the presence of the nearby primary ICR (primary “IG-DMR” and secondary “*Meg3* DMR” in the *Dlk1-Dio3* domain) [4–7]. Loss of the maternal *Igf2-H19* ICR or mutations in its CTCF binding sites lead to the adoption of the paternal transcriptional program, indicating an essential role for allelic CTCF binding [8, 9].

The CTCF insulator protein is essential for the organization of the genome into “Topologically Associating Domains” (TADs) [10–12]. TADs are 3D structures with enriched intra-domain interactions that tend to insulate genes and their regulatory elements [13]. TAD borders are enriched for CTCF binding sites, with a strong enrichment for convergent sites located at both sides of the TAD [10, 14]. Disruption of CTCF binding sites at certain, but not all, TAD borders leads to inappropriate activation of surrounding genes during development [15, 16]. Within TADs, further levels of chromatin organization can be observed, sometimes referred to as sub-TADs, with CTCF often being implicated as well [17, 18].

The reported allele-specific binding of CTCF at the DMRs of the paternally imprinted *Igf2-H19* and *Dlk1-Dio3* domains urged us to investigate the chromatin structure of these domains within the context of TAD organization. Previously, non-comprehensive 3C (“Chromosome Conformation Capture”) studies at the *Igf2-H19* domain reported various instances of allele-specific chromatin looping ([19–23], see the “Discussion” section for details). Yet, how these loops are embedded within (sub-)TADs remains unknown due to the incomplete views of DNA contacts and CTCF binding. Moreover, whether the *Dlk1-Dio3* domain adopts a similar allelic 3D architecture, and how chromatin structure is reorganized during imprinted gene activation, remains unexplored. Here, we combined studies of allelic CTCF binding with both high-resolution and single-cell 3D chromatin organization assays to determine the dynamic structuration of the paternally imprinted *Igf2-H19* and *Dlk1-Dio3* domains. Moreover, for the less-characterized *Dlk1-Dio3* domain, we performed mechanistic studies to demonstrate the structural and functional importance of allele-specific CTCF binding for correct imprinted gene activation during cellular differentiation.

## Results

### The *Igf2-H19* and *Dlk1-Dio3* domains are located in TADs that include multiple sites of mono- and bi-allelic CTCF binding

**Fig. 1:**
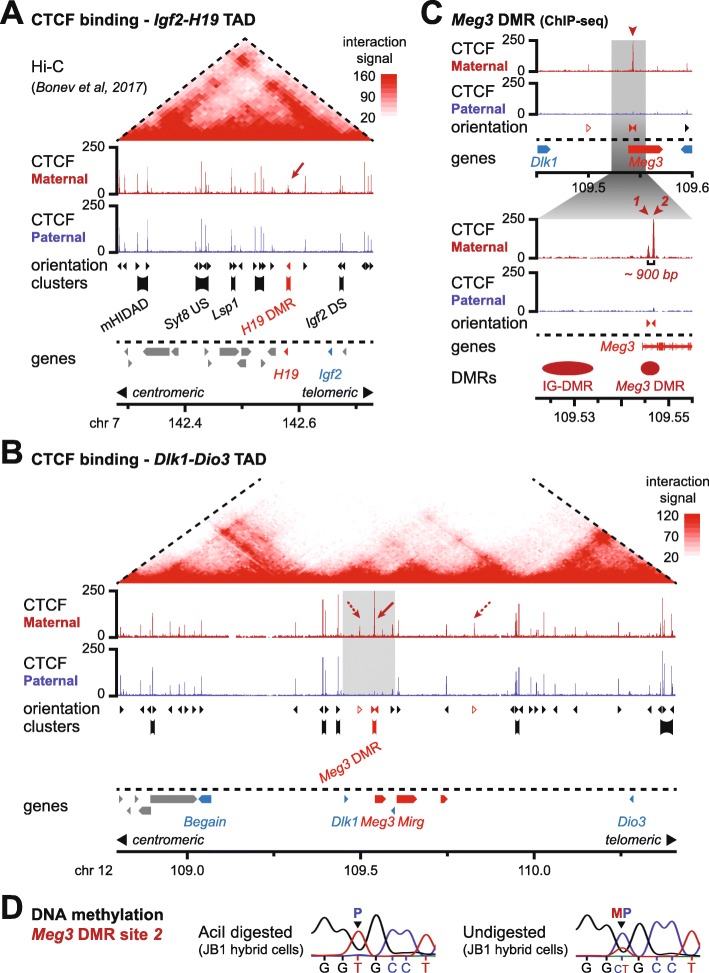
Multiple instances of bi-allelic CTCF binding accompany maternal-specific CTCF binding at the *Meg3* and *H19* DMRs. **a** CTCF ChIP-seq signal in the TAD containing the *Igf2-H19* domain on the maternal (red) and paternal (blue) chromosome in mono-parental ESCs. The arrow highlights maternal-specific CTCF binding at the *H19* DMR. Reanalyzed Hi-C signal is indicated above ([11]; 10 kb resolution); the dotted lines indicate TAD boundaries (TADtool [24]). The orientation of CTCF sites, extent of CTCF clusters, genes, and centromeric and telomeric orientation are indicated below with colors indicating allele specificity. **b** CTCF ChIP-seq signal in the TAD containing the *Dlk1-Dio3* domain on the maternal (red) and paternal (blue) chromosome in mono-parental ESCs. The arrow highlights maternal-specific CTCF binding at the *Meg3* DMR. Gray box indicates the region covered by the zoom-in in **c**. Reanalyzed Hi-C signal is indicated above [11]; the dotted lines indicate TAD boundaries. **c** Sequential zoom-in on the region around the IG-DMR and *Meg3* DMR reveals the CTCF peak at the *Meg3* DMR separates into two sub-peaks that are 900 bp apart (sites 1 and 2). The location of the *Meg3* transcript and the IG-DMR and *Meg3* DMR are indicated below. **d** Confirmation of maternal-specific DNA methylation at the *Meg3* DMR in JB1 hybrid ESCs by Sanger sequencing of genomic DNA with (top) and without (bottom) methylation-sensitive *AciI* digestion. The parental origin of a SNP that distinguishes the maternal and paternal alleles is indicated

To investigate how the *Igf2-H19* and *Dlk1-Dio3* domains are embedded within their respective TADs, we reanalyzed high-resolution, but non-allelic, Hi-C data in ESCs [11]. This analysis positioned the *Igf2-H19* and *Dlk1-Dio3* domains within TADs of about 450 kb and 1.6 Mb, respectively (Fig. 1a, b). To address if a parent-of-origin bias may be introduced by allele-specific CTCF binding in these TADs, we performed ChIP-seq on ground-state parthenogenetic (PR8) and androgenetic (AK2) embryonic stem cells (ESCs). For the *Igf2-H19* domain, we detected maternal allele-specific binding of CTCF within the TAD only at the well-characterized ICR located 2–4 kb towards the telomeric side from the *H19* gene (Fig. 1a, arrow, and Additional file 1: Figure S1a). At the *Dlk1-Dio3* domain, our ChIP-seq analysis did not detect CTCF binding at the primary ICR (“IG-DMR”). In contrast, we identified three instances of putative allelic CTCF binding in the TAD on the maternal chromosome (Fig. 1b, arrows in maternal ChIP-seq track; see Fig. 1c for location of relevant DMRs). ChIP-seq validation was performed in ESCs (line “BJ1”) that were hybrid between the *M. m. domesticus* C57BL/6 and *M. m. molossinus* JF1 inbred lines. This confirmed maternal allele-specific CTCF binding only at the most prominent of these three putative sites (Fig. 1b, solid arrow, and Additional file 2), which we retained for further analysis. This site overlaps the previously identified maternal-specific CTCF binding in humans and mouse [5, 6], and is located in the secondary *Meg3* DMR that covers the promoter and the first intron of the maternally expressed *Meg3* lncRNA (Fig. 1c and Additional file 3). Closer inspection of this site in the mouse revealed it separated into two peaks that are 900 bp apart (Fig. 1c; sites 1 and 2). ChIP-qPCR experiments in the mono-parental and hybrid ESCs confirmed the robust enrichment of CTCF binding on the maternal chromosome at this site (Additional file 1: Figure S1b). Methylation-sensitive endonuclease digestion showed that this intronic site is methylated on the paternal chromosome, both in hybrid ESCs and in day 9.5 embryos (E9.5) (Fig. 1d and Additional file 1: Figure S1c, d). Both the paternally imprinted domains therefore include allele-specific CTCF recruitment, on the non-methylated maternal allele only, either at the primary ICR itself (*H19* DMR at the *H19-Igf2* domain) or at a close-by secondary DMR (*Meg3* DMR at the *Dlk1-Dio3* domain).

An uncharacterized aspect of both the paternally imprinted domains is extensive additional CTCF binding within their overarching TADs. Our ChIP-seq analysis detected multiple instances of bi-allelic CTCF binding at both the *Igf2-H19* and the *Dlk1-Dio3* domain (Fig. 1a, b). Within the 450-kb TAD that contains the *Igf2-H19* domain, we particularly noticed the presence of 4 centromeric clusters of bi-allelic CTCF binding, positioned between 50 and 250 kb away from the *H19* ICR (Fig. 1a). At the much larger, 1.6 Mb, *Dlk1-Dio3* TAD, we noticed multiple “patches” of bi-allelic CTCF binding as well, including 2 noticeable clusters of CTCF binding located about 150 kb towards the centromeric side of the maternal-specific CTCF-bound DMR (Fig. 1b).

In conclusion, both the *Igf2-H19* and the *Dlk1-Dio3* domains are characterized by a single element carrying allelic CTCF binding, at either the maternal *H19* DMR or *Meg3* DMR, combined with multiple sites of clustered intra-TAD bi-allelic CTCF binding. These combinations of bi-allelic and maternal allele-specific CTCF binding sites may structure TAD organization at both the domains, or further levels of sub-TAD organization within.

### The *Igf2-H19* and *Dlk1-Dio3* domains are located within TADs that do not exhibit allelic differences, as measured by allele-specific 4C-seq

First, we wondered whether the maternal-specific CTCF binding accompanied differences in overall TAD structure at both the paternally imprinted domains. To address this question, we performed high-resolution allele-specific 4C-sequencing using multiple viewpoints in both the *Igf2-H19* and *Dlk1-Dio3* domains (Additional file 1: Figure S2a, b). As expected, 4C-seq signal obtained for all viewpoints in the domains was largely confined to the TAD that contained the viewpoints themselves, and little signal was detected in neighboring TADs (Additional file 1: Figure S2c, d). More importantly, a quantitative comparison between the maternal and paternal chromosomes revealed highly similar signal distributions (Additional file 1: Figure S2e). We conclude that maternal allele-specific CTCF binding at the *Igf2-H19* and *Dlk1-Dio3* domains does not result in structural changes at the level of overarching TADs.

### Allele-specific sub-TAD organization of the *Igf2-H19* imprinted domain

**Fig. 2:**
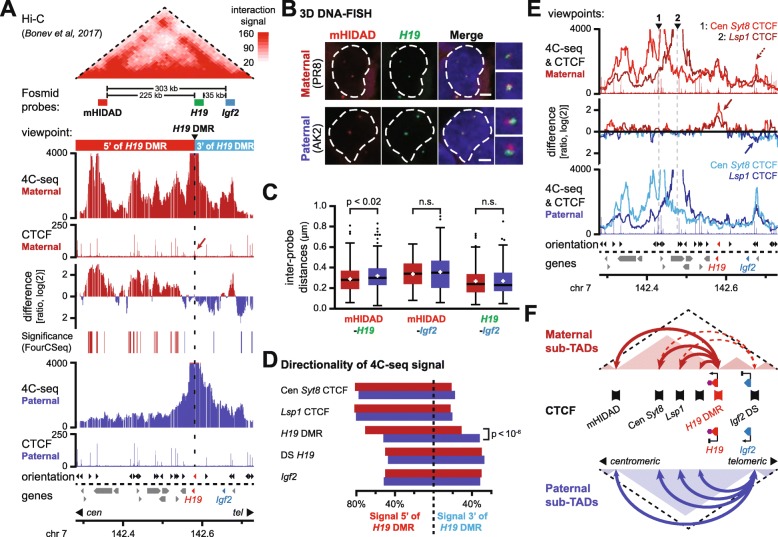
Allele-specific sub-TAD organization of the *Igf2-H19* domain. **a** 4C-seq signal for the *H19* DMR viewpoint in the *Igf2-H19* TAD on the maternal (red) and paternal (blue) chromosome in mono-parental ESCs. The ratio of maternal/paternal interactions is provided in between, with significant differences in both replicates, as determined by FourCSeq, indicated below [25]. CTCF ChIP-seq signal is indicated below, with the arrow pinpointing the *H19* DMR. Reanalyzed Hi-C signal [11], fosmid probes, the position of the viewpoint, and span of maternal sub-domains are indicated above. **b** Examples of 3D DNA-FISH with fosmid probes in the *Igf2-H19* TAD (see **a**). Images show representative cells in mono-parental ESCs. Scale bars, 2 μm. **c** 3D DNA-FISH distance measurements in mono-parental ESCs reveal a smaller distance between the mHIDAD and *H19* probes on the maternal chromosome. **d** Directionality of 4C-seq signal for indicated viewpoints in the 5′ and 3′ sub-domains (see **a**). **e** 4C-seq line graphs for two bi-allelic CTCF clusters (viewpoint 1: cluster centromeric from the *Syt8* gene; viewpoint 2: cluster in *Lsp1* gene). Arrows indicate increased allele-specific signal at the *H19* DMR (maternal) or telomeric from the *Igf2* gene (paternal). **f** Schematic representation of allele-specific CTCF-structured sub-TAD organization at the *Igf2-H19* domain. CTCF clusters (banners), allele specifically expressed genes (triangles), and reported regulatory elements (hexagon) are indicated

To determine if maternal allele-specific CTCF binding may reorganize chromatin organization at the sub-TAD level, we next reassessed our 4C-seq data for the well-characterized *Igf2-H19* domain (Additional file 1: Figure S2a). On the maternal chromosome—both in mono-parental and hybrid ESCs—the *H19* DMR interacted significantly more with all four centromeric clusters of bi-allelic CTCF binding than its paternal counterpart (Fig. 2a and Additional file 1: Figure S3a, b). In agreement with the orientation of CTCF binding at the *H19* DMR, all interacting clusters contained at least one binding site orientated towards the *H19* DMR. In contrast, a different configuration was detected at the paternal chromosome, where interactions were globally increased around the viewpoint and towards the 3′ of the *H19* DMR.

To explore whether the allelic DNA interactions within the *Igf2-H19* TAD correlate with differential higher-order configuration, we measured inter-probe distances by 3D DNA-FISH using fosmid probes covering *H19*, *Igf2*, or mHIDAD, the mouse homologue of the bi-allelic CTCF-bound “*H19*/*Igf2* Distal Anchor Domain” [14] (Fig. 2a, b). Consistent with our 4C-seq results, the average distance between the *H19* and mHIDAD probes was significantly smaller on the maternal chromosome (Fig. 2c and Additional file 1: Figure S3c). Intriguingly though, the distances between the *Igf2* and mHIDAD probes were not significantly different on the maternal and paternal chromosomes. Similarly, we did not detect allelic differences in distance between the *H19* and *Igf2* probes (Fig. 2c and Additional file 1: Figure S3c). Consistently, a 4C-seq viewpoint containing the *Igf2* promoter showed no major allelic differences in its intra-TAD interactions either (Additional file 1: Figure S3d). Further reinforcing these observations, statistical analysis of the 4C-seq data confirmed that the parental distribution of signal within the subdomains 5′ and 3′ of the *H19* DMR was significantly different only for the *H19* DMR viewpoint (*G* test; Fig. 2d and Additional file 1: Table S1a). Although these results may initially appear incompatible (see the “Discussion” section), we conclude that the CTCF-anchored loops on the maternal chromosome structure a sub-TAD organization that increases the insulation between the reported regulatory elements near the *H19* DMR and the more telomeric *Igf2* gene [4, 26].

To get a better appreciation of global sub-TAD structure on both the parental chromosomes, we performed further 4C-seq on two of the centromeric bi-allelic CTCF clusters (*Syt8* US and *Lsp1* clusters). Whereas both clusters looped towards the *H19* DMR on the maternal chromosome, they formed chromatin loops on the paternal chromosome as well, but now with the more distal bi-allelic CTCF cluster located telomeric from the *Igf2* gene (Fig. 2e, arrows, and Additional file 1: Figure S3e). In the absence of maternal CTCF binding to the *H19* DMR, these centromeric-located CTCF binding clusters thus extended their intra-TAD loops towards further telomeric-located CTCF sites that are bi-allelic in nature as well.

On both the parental chromosomes, the TAD containing the *Igf2-H19* domain therefore has a specific sub-TAD configuration consisting of ensembles of CTCF-anchored chromatin loops. Within this configuration, allele-specific differences are implemented only by CTCF binding at the maternal *H19* DMR (Fig. 2f).

### Recruitment of CTCF to the maternal *Meg3* DMR structures a localized *Dlk1-Meg3* sub-TAD

**Fig. 3:**
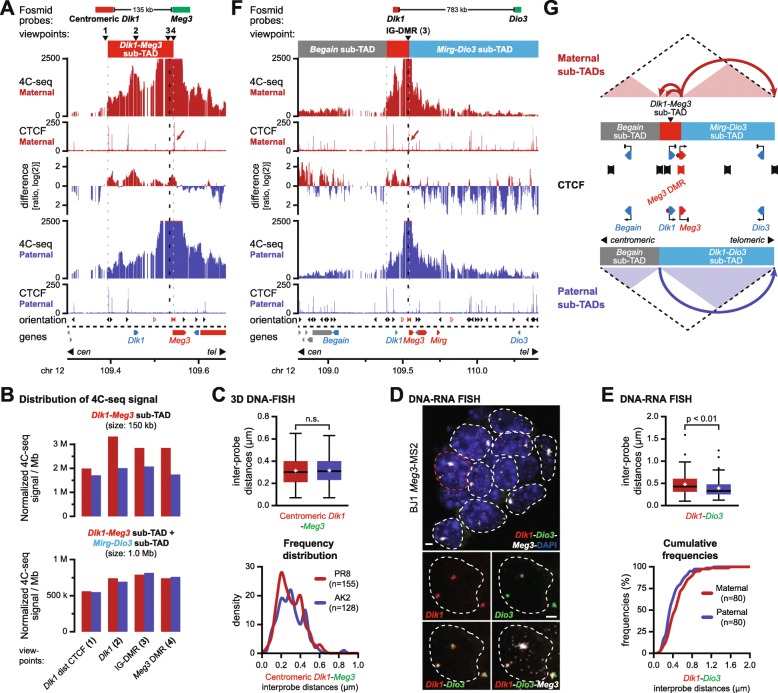
Allele-specific sub-TAD organization of the *Dlk1-Dio3* domain. **a** 4C-seq signal for the IG-DMR viewpoint in a 300-kb region around *Dlk1-Meg3* on the maternal (red) and paternal (blue) chromosome in hybrid ESCs. The ratio of maternal/paternal interactions is provided in between. CTCF ChIP-seq signal is indicated below, with the arrow pinpointing the *Meg3* DMR. Fosmid probes, positions of the viewpoints (see **b**), and the span of the maternal *Dlk1-Dio3* sub-TAD are indicated above. **b** Distribution of 4C-seq signal for indicated viewpoints in the *Dlk1-Meg3* sub-TAD (top) and the combined *Dlk1-Meg3* and *Mirg-Dio3* sub-TADs (bottom). **c** 3D DNA-FISH distance measurements with fosmid probes (see **a**) reveal similar distances between both sides of the *Dlk1-Meg3* sub-TAD in mono-parental ESCs. **d** Allele-specific DNA-RNA FISH with combined fosmids and *Meg3* RNA probes (MS2 sequences) in hybrid ESCs. Images show representative cells. Scale bars, 2 μm. **e** DNA-RNA FISH distance measurements (see **f**) reveal a larger distance between *Dlk1* and *Dio3* on the maternal chromosome. **f** 4C-seq signal for the IG-DMR viewpoint across the entire *Dlk1-Dio3* TAD. Fosmid probes and the span of the sub-TADs (red box: *Dlk1-Meg3* sub-TAD; see **a**) are indicated above. **g** Schematic representation of allele-specific CTCF-structured sub-TAD organization at the *Dlk1-Dio3* domain. CTCF clusters (banners), allele specifically expressed genes (triangles), and reported regulatory elements (hexagons) are indicated

Next, we shifted our attention to the less characterized *Dlk1-Dio3* domain, which resides within a 1.6-Mb TAD (Fig. 1b). To assess the potential involvement of the maternal allele-specific CTCF binding at the *Meg3* DMR, we reassessed our 4C-seq viewpoints targeting both the *Meg3* DMR and the germline ICR (“IG-DMR”) that acts as a maternal-specific *Meg3* enhancer [27], as well as the *Dlk1* gene and the CTCF binding site centromeric from the *Dlk1* gene, both in hybrid and mono-parental ESCs (Additional file 1: Figure S1a). Consistently, all four viewpoints showed significantly increased interactions on the maternal chromosome, within a 150-kb domain that is demarcated on the right side by the maternal-specific CTCF sites 1 and 2 and on the left by the two bi-allelic clusters of convergently oriented CTCF clusters (Fig. 3a, b, and Additional file 1: Figure S4a and Table S1b). This maternal sub-TAD, which we named the *Dlk1-Meg3* sub-TAD, contains the promoters of *Dlk1* and *Meg3*, both the IG-DMR and the *Meg3* DMR, and several putative *Dlk1* regulatory elements [28]. Like for the maternal *Igf2-H19* sub-domain (Fig. 2), the structure of the *Dlk1-Meg3* sub-TAD involves both maternal- and bi-allelic CTCF sites.

To address how the presence of the *Dlk1-Meg3* sub-TAD correlated with spatial distances between imprinted genes, we performed DNA-FISH using fosmid probes at the borders of this small sub-domain. Inter-probe distances were on average similar between the parental chromosomes (Fig. 3a, c, and Additional file 1: Figure S4b). This finding is similar to our observation that most DNA-FISH distances within the imprinted *Igf2-H19* domain are not different between the two parental chromosomes.

Next, we determined how the presence of the *Dlk1-Meg3* sub-TAD on the maternal chromosome influenced contacts between other regions of the imprinted domain. We assayed potential allelic differences in higher-order domain organization by 3D DNA-FISH in both hybrid and mono-parental ESCs (Fig. 3d, e, and Additional file 1: Figure S5a-d). Interestingly, significantly longer distances were measured on the maternal chromosome between all pairs of DNA-FISH probes that covered the *Dlk1*, *Dio3*, and *Begain* genes. Therefore, the regions within the TAD that contain the three protein genes that are inactive in ESCs but may acquire paternal allele-specific expression upon differentiation (Additional file 3 and [29, 30]) are more frequently positioned in closer proximity on the paternal chromosome.

To gain further insight into these differential configurations, we performed 4C-seq in mono-parental ESCs using viewpoints near the *Dlk1*, *Dio3*, and *Begain* promoters (Additional file 1: Figure S5e). Replicated experiments revealed no consistent differences between the maternal and paternal chromosomes, including no specific long-range intra-TAD DNA loops on either chromosome beyond the presence of the *Dlk1-Meg3* sub-TAD for the *Dlk1* viewpoint (see also Additional file 1: Figure S4a), and a moderate non-allelic enrichment in interactions between the H3K27me3-marked promoters in the domain [31] (Additional file 1: Figure S5e, asterisks).

Finally, we determined if the maternal *Dlk1-Meg3* sub-TAD itself engaged in differential contacts with other regions in the TAD. For this purpose, we further analyzed our four 4C-seq viewpoints in the *Dlk1*-*Meg3* sub-TAD (Additional file 1: Figure S2a) for differential contacts within the remainder of the TAD. In the centromeric sub-domain, which we termed the “*Begain* sub-TAD,” no major difference in total 4C-seq signal between the parental chromosomes was observed. In contrast, in the telomeric sub-domain, which we named the “*Mirg-Dio3* sub-TAD,” 4C-seq signal for all four viewpoints was consistently increased on the paternal allele (Fig. 3f and Additional file 1: Figure S4a and S5f). Contacts in the *Mirg-Dio3* sub-TAD therefore displayed an opposite pattern as compared to the maternally enriched *Dlk1-Meg3* sub-TAD. Whereas this trend was highly significant between the two sub-TADs, their combined signal was essentially the same on the parental chromosomes (Fig. 3b and Additional file 1: Table S1c). CTCF binding at the maternal chromosome therefore changed the distribution of 4C-seq contacts between the *Dlk1-Meg3* and *Mirg-Dio3* sub-TADs, rather than imposing a complete restructuration of chromatin architecture. This increased insulation may explain the particularly long average DNA-FISH distances on the maternal allele between the *Dio3* probe and probes located in the *Dlk1-Meg3* sub-TAD (Fig. 3e and Additional file 1: Figure S5b, d).

In summary, our combined 4C-seq and DNA-FISH studies in ESCs revealed that the *Dlk1-Dio3* TAD is organized into sub-domains that manifest themselves at multiple levels. The paternal chromosome is structured into the *Begain* sub-TAD and the *Dlk1-Dio3* sub-TAD. On the maternal chromosome, allele-specific CTCF binding at the *Meg3* DMR further divides the *Dlk1-Dio3* sub-domain into the *Dlk1-Meg3* and *Mirg-Dio3* sub-TADs, without affecting the presence of the *Begain* sub-TAD (Fig. 3g). In contrast to the relatively small *Igf2-H19* domain and its host TAD, maternal CTCF binding at the much larger *Dlk1-Dio3* domain and TAD structures a localized sub-domain (*Dlk1-Meg3*), whose insulated nature coincides with an increased average spatial distance between the *Begain*, *Dlk1*, and *Dio3* genes on the maternal chromosome (Fig. 3e and Additional file 1: Figure S5a-d).

### CTCF binding at the *Meg3* DMR is required for allelic sub-TAD structuration and for correct imprinted activation of *Dlk1*

**Fig. 4:**
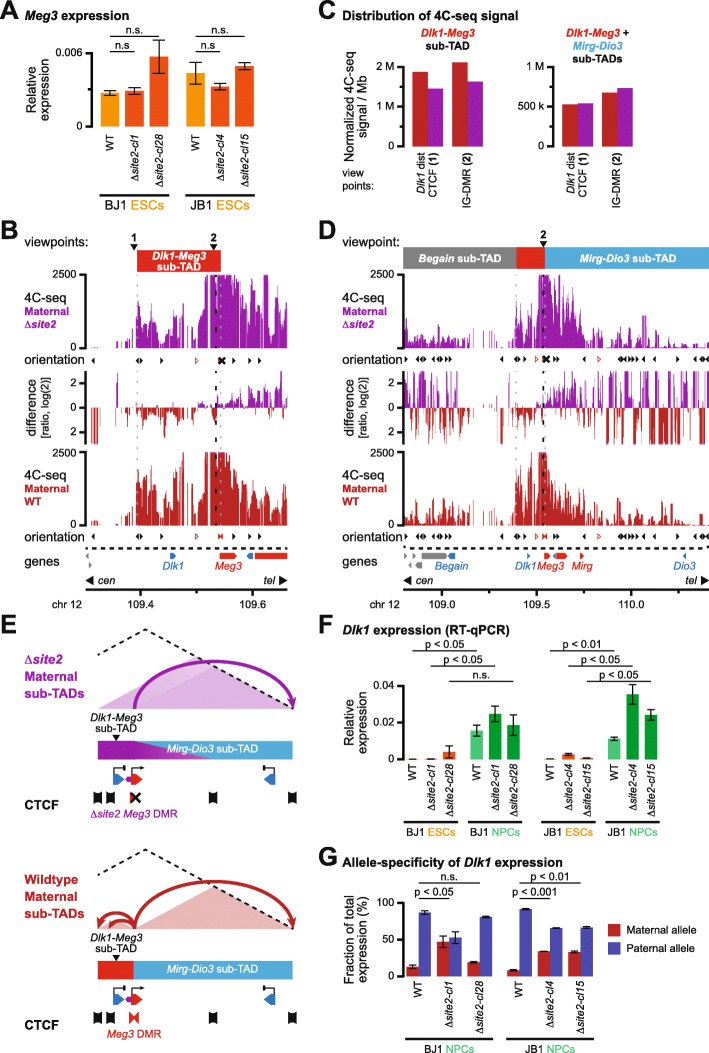
Allelic CTCF binding at the *Meg3* DMR is essential for correct sub-TAD organization. **a** *Meg3* expression in hybrid ESCs is maintained upon deletion of CTCF binding site 2 in the *Meg3* DMR, as determined by qRT-PCR. Error bars indicate standard error of the mean (SEM), with significance of difference determined using a two-sided unpaired *t* test (*n* = 2). n.s., not significant. **b** 4C-seq signal for the IG-DMR (viewpoint 2) on the maternal alleles in hybrid ESCs with a deleted CTCF site 2 in the *Meg3* DMR (purple) or their WT counterparts (red) in a 300-kb region around the *Dlk1-Dio3* DMRs. The ratio of interactions is provided in between. The orientation of CTCF sites is indicated below each panel, with the X indicating the deleted CTCF site. Viewpoints and the maternal *Dlk1-Dio3* sub-TAD are indicated above. **c** Distribution of 4C-seq signal for indicated viewpoints in the *Dlk1-Meg3* sub-TAD (left) and the combined *Dlk1-Meg3* and *Mirg-Dio3* sub-TADs (right). **d** 4C-seq signal for the IG-DMR viewpoint across the entire *Dlk1-Dio3* TAD. The position of the sub-TADs (red box: *Dlk1-Meg3* sub-TAD) is indicated above. **e** Schematic depiction of the maternal *Dlk1-Meg3* and *Mirg-Dio3* sub-TAD reorganization upon deletion of CTCF binding site 2 in the *Meg3* DMR. CTCF clusters (banners), allele specifically expressed genes in normal cells (triangles), and reported regulatory elements (hexagons) are indicated. **f** *Dlk1* expression levels in hybrid ESCs and in vitro differentiated NPCs with a deleted CTCF site 2 in the *Meg3* DMR and their WT counterparts, as determined by qRT-PCR. Error bars indicate SEM, with significance of difference determined using a two-sided unpaired *t* test (*n* = 2). **g** Allelic *Dlk1* expression becomes relaxed in hybrid NPCs carrying a deletion in CTCF binding site 2 in the *Meg3* DMR, as determined by qRT-PCR. Error bars indicate SEM, with significance of difference between the maternal alleles determined using a one-sided unpaired *t* test (*n* = 2)

To confirm if CTCF binding to the maternal *Meg3* DMR is essential for the structure of the maternal *Dlk1-Meg3* sub-TAD and for correct imprinted activation of the *Dlk1* gene, we perturbed CTCF binding using CRISPR-Cas9 genome editing (Additional file 1: Figure S6a). The *Meg3* DMR contains two CTCF binding sites that are 900 bp apart (sites 1 and 2, Fig. 1c). We focused on CTCF binding site 2, where CTCF binding is most prominent and conserved in humans [5], and which is oriented towards the CTCF clusters at the 5′ side of the *Dlk1-Meg3* sub-TAD. Multiple clonal lines with short bi-allelic deletions comprising site 2 were obtained in both BJ1 and (JF1 × C57BL/6 J)F1 mESC (line JB1, Additional file 1: Figure S6a). The deletion lines displayed correct imprinted expression and unaltered nuclear accumulation of the *Meg3* lncRNA (Fig. 4a and Additional file 1: Figure S6b, c), and unaltered ESC morphology and growth (not shown). Moreover, correct CTCF binding at site 1 and expression of other non-coding RNAs in the domain were maintained in the further characterized hybrid JB1 Δ*site2-cl4* ESCs clone (Additional file 1: Figure S6d, e).

To determine if allelic CTCF binding directly determined the formation of the *Dlk1-Meg3* sub-TAD, we performed 4C-seq on the JB1 Δ*site2-cl4* ESCs clone. Using viewpoints at the IG-DMR and the CTCF peak centromeric of the *Dlk1* gene, we noted that absence of maternal CTCF binding at site 2 strongly reduced interactions within the *Dlk1-Meg3* sub-TAD on the maternal chromosome (Fig. 4b, c, and Additional file 1: Figure S6f). Conversely, within the *Mirg-Dio3* sub-TAD, maternal interactions were increased in the mutant cells. Yet, interactions within both sub-TADs combined remained constant (Fig. 4c, d, and Additional file 1: Figure S6f, g). Based on these results, we conclude that the maternal *Dlk1-Meg3* sub-TAD in the absence of CTCF binding at *Meg3* DMR site 2 adopts a more paternal-like 3D configuration (Fig. 4e).

In normal WT cells, the domain’s imprinted protein-coding genes, particularly *Dlk1*, are activated upon neural differentiation from the paternal allele [29, 30]. To explore if the perturbation of the *Dlk1-Meg3* sub-TAD structure leads to incorrect imprinted activation of the *Dlk1* gene, we performed in vitro differentiation of our WT and Δ*site2* hybrid (BJ1 and JB1) ESCs into neural progenitor cells (NPCs) with cortical identity. In this system, imprinted *Dlk1* activation can be readily recapitulated [30, 32]. The NPCs differentiated from our JB1 and BJ1 deletion ESC lines were developmentally comparable to WT ESC-derived NPCs (Additional file 1: Figure S7a). In all NPC lines, *Meg3* remained strictly expressed from the maternal chromosome (Additional file 1: Figure S7b, c). Moreover, in the further characterized hybrid JB1 Δ*site2-cl4* ESCs clone, methylation-sensitive endonuclease digestion showed the expected 50% methylation level at both the IG-DMR and the *Meg3* DMR (Additional file 1: Figure S7d). As in WT cells, *Dlk1* expression strongly increased upon neural differentiation in our Δ*site2* hybrid cells (Fig. 4f). Deletion of CTCF binding at the *Meg3* DMR site 2 therefore did not interfere with differentiation into NPCs, with the overall paternally imprinted DNA methylation status, with persistence of maternal *Meg3* expression or with *Dlk1* activation.

In contrast, in all deletion lines, we observed increased transcriptional activation not only from the paternal chromosome, but also from the maternal chromosome. Allele-specific qRT-PCR revealed that on average, 31% of total *Dlk1* transcripts in our deletion NPC lines were of maternal origin (Fig. 4g and Additional file 1: Figure S7c). In the absence of CTCF binding to site 2 in the *Meg3* DMR, imprinted *Dlk1* expression therefore becomes partially relaxed. We conclude that CTCF binding to site 2 in the *Meg3* DMR, which is essential for the correct structure of the *Dlk1-Meg3* sub-TAD, prevents activation of the *Dlk1* gene from the maternal chromosome during neural differentiation.

### *Dlk1* activation upon neural differentiation occurs without major restructuring of sub-TAD organization

**Fig. 5:**
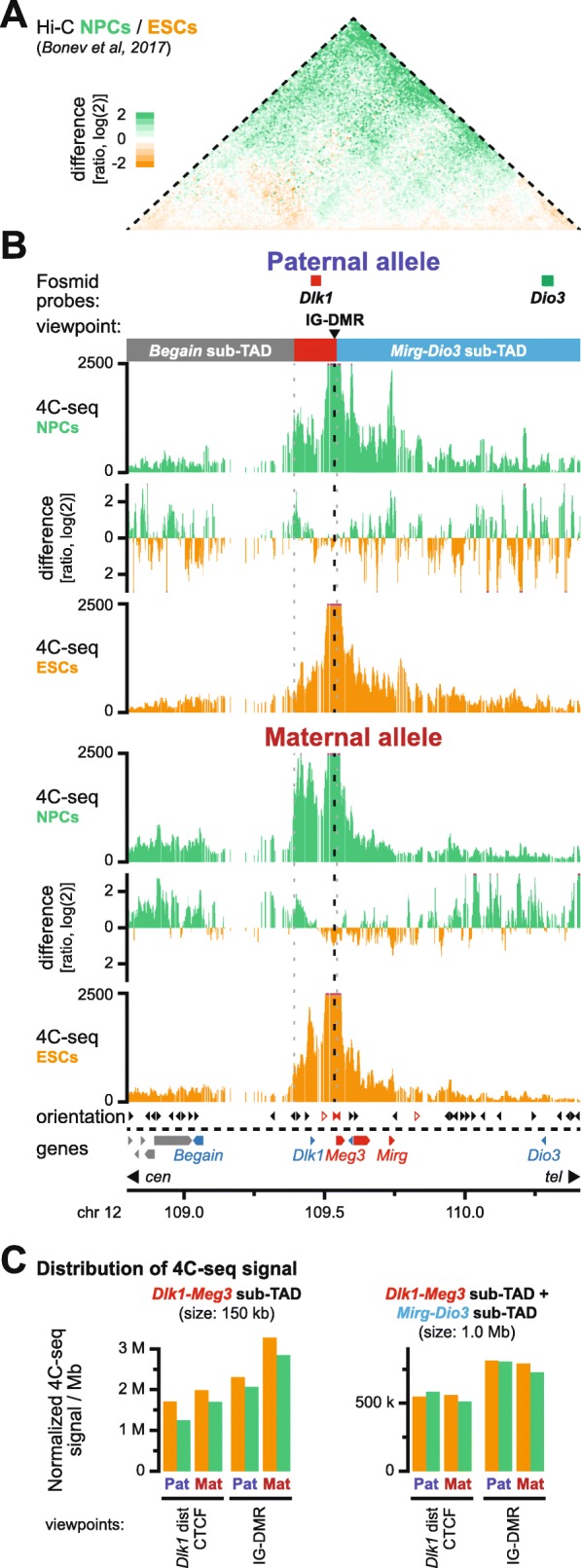
Paternal *Dlk1* activation during neural differentiation occurs without major intra-TAD reorganization. **a** Differential non-allelic Hi-C signal upon differentiation of ESCs (orange) to NPCs (green). Hi-C data from reference [11]. **b** 4C-seq signal for the IG-DMR viewpoint in in vitro differentiated hybrid NPCs and ESCs on the paternal (top) and maternal (bottom) chromosome in the *Dlk1-Dio3* TAD. The positions of fosmid probes, the viewpoint, and the sub-TADs (red box: *Dlk1-Meg3* sub-TAD) are indicated above. **c** Distribution of 4C-seq signal for indicated viewpoints in the *Dlk1-Meg3* sub-TAD (left) and the combined *Dlk1-Meg3* and *Mirg-Dio3* sub-TADs (right)

To explore whether the allelic, CTCF-associated sub-TAD organization could contribute directly to the lack of *Dlk1* activation on the maternal chromosome, we assessed whether it is maintained in the in vitro differentiated NPCs. Re-analysis of published non-allelic Hi-C data, using the same in vitro differentiation protocol of ESCs into NPCs, revealed a similar chromatin architecture of the *Dlk1-Dio3* TAD, with only—as previously reported—a minor increase in long-range intra-TAD interactions upon in vitro differentiation [11] (Fig. 5a, green shading, and Additional file 1: Figure S8a). We performed allelic 4C-seq on ESC-derived hybrid NPCs using viewpoints at the IG-DMR and the bi-allelic CTCF peak centromeric of the *Dlk1* gene (Fig. 5b and Additional file 1: Figure S8b, c). Similar to ESCs, we observed allele-specific differences in the distribution of 4C-seq signal between the *Dlk1-Meg3* and *Mirg-Dio3* sub-TADs, with the total amount of signal in the combined sub-TADs being similar between the parental chromosomes (Fig. 5c, Additional file 1: Figure S8d and Table S1d). In vitro differentiation was therefore not accompanied by drastic reorganization of sub-TAD configuration. Direct comparison between NPCs and ESCs, on either the paternal or maternal chromosome, revealed no major domain-wide changes in chromatin contacts either (Fig. 5b and Additional file 1: Figure S8c). Moreover, comparison of 3D distances between *Dlk1* and *Dio3* using DNA-FISH, although non-allele specific, revealed no significant change in the relative distance between sub-TADs upon differentiation either (Additional file 1: Figure S8e). Any changes detected in the 4C-seq analysis therefore do not represent a major reorganization of sub-TAD configuration between NPCs and ESCs. Rather, we conclude that the *Dlk1-Dio3* sub-TAD organization remains mostly stable during differentiation, which may thus provide essential allele-specific scaffolding that is required for the correct imprinted activation of *Dlk1* during stem cell differentiation.

## Discussion

In this study, we dissected chromatin structure of the two conserved paternally imprinted domains—*Igf2-H19* and *Dlk1-Dio3*—and their surrounding TADs, using genomics and imaging-based approaches. Both domains show maternal-specific CTCF binding at DMRs, together with multiple sites of bi-allelic CTCF binding, to structure localized maternal allele-specific sub-TADs. Unlike the allele-specific chromosome organization in X-chromosome inactivation [14, 33], these sub-structures are contained within overarching TADs that are similar on both the parental chromosomes. Rather, at both imprinted domains, maternal allele-specific CTCF binding hijacks an existing TAD organization that is formed between bi-allelic CTCF bound clusters. The resulting maternal chromosome-specific sub-TADs are already established in ESCs, before imprinted activation of protein-coding genes on the paternal alleles [30]. These allele-specific sub-TADs may thus provide the “instructive” or “permissive” context for correct developmentally regulated imprinted gene expression during development [34].

More generally, our study highlights striking mechanistic similarities between the two paternally imprinted domains conserved in mammals. At both the domains, sperm-derived DNA methylation imprints mediate, directly or indirectly, maternal allele-specific binding of CTCF to key regions. This, in turn, is accompanied by the presence of sub-TADs that prevent the developmental activation of essential protein-coding genes (*Igf2* at *H19-Igf2*, and *Dlk1* at *Dlk1-Dio3*) from the maternal chromosome. Perturbation of maternal CTCF binding at the DMRs results in loss of imprinting of the paternally expressed protein-coding genes, as determined in this study (*Meg3* DMR) or previously published (*H19* DMR) [8]. Although the maternal sub-TADs at both paternally imprinted domains are directly associated with lncRNA expression, these sub-TADs may thus ultimately have evolved within a framework of overarching non-allelic TADs [35] to repress the developmentally regulated activation of the paternally expressed genes by overriding the inherent action of the paternal 3D organization.

Comparison of our newly identified allele-specific sub-TAD architecture of the *Igf2-H19* domain to previously reported non-comprehensive allele-specific 3C and CTCF-binding data provides both further support for our model and important nuance to the interpretation of these previous studies. Using 3C, a considerable number of allele-specific DNA loops between the regulatory elements (promoters, enhancers, DMRs) on both the maternal and paternal chromosomes were reported in a region spanning 150 kb around the *H19* and *Igf2* genes [19–23]. Careful mapping of these previous 3C results within the context of our newly described sub-TAD organization reveals that these studies have in common that 3C contacts on the maternal chromosome are limited to either side of the CTCF-bound *H19* DMR, whereas on the paternal chromosome, contacts can also surpass the element (Additional file 1: Figure S9). Rather than measuring specific allele-specific chromatin loops at the domain, we therefore consider it more likely that these 3C studies measured the insulated nature of the two maternal sub-domains that are separated by the *H19* DMR, which is in agreement with our 4C-seq-based analysis.

How the observed sub-TAD structuration achieves gene repression *in cis* is different between the two domains though. At the *Igf2-H19* domain, the maternal sub-TADs increase the insulation between the *Igf2* gene and enhancers that can activate both *Igf2* and *H19* [26] (Fig. 2f). This mechanism is further supported by a previous study, where positioning of the enhancers in the maternal sub-TAD that contains the *Igf2* gene resulted in the inversion of imprinted gene activation [36]. As such, these sub-TADs function as “instructive” chromosomal neighborhoods that delineate gene-enhancer contacts [15, 34]. Yet, their presence or absence is tuned through an epigenetic switch within the context of normal developmental and parental origin.

In contrast, at the *Dlk1-Dio3* domain, the CTCF-structured maternal *Dlk1*-*Meg3* sub-TAD further clusters the repressed *Dlk1* gene and its identified regulatory elements (Fig. 3g). The necessity of CTCF binding to the *Meg3* DMR, associated with the maternal-specific clustering of the sub-TAD, was shown by deletion of CTCF binding site 2, which resulted in a more relaxed imprinting of the *Dlk1* gene, with developmental activation now occurring on both parental chromosomes. Whether presence of the maternal *Dlk1-Meg3* sub-TAD may similarly repress the imprinted activation of the *Begain* and *Dio3* genes remains to be determined. Both genes are located at considerable distance within the large TAD, and within different sub-TADs (Fig. 3g). These genes do not become activated upon in vitro neuronal differentiation and are not imprinted in in vitro generated cortical NPCs, complicating the study of their imprinted activation [30, 32]. Recently, we found that the maternal expression of *Meg3*, and possibly the lncRNA itself, prevents activation of *Dlk1* as well [30]. We hypothesize that the two mechanisms are linked, with either the *Dlk1-Meg3* sub-TAD focusing or constraining the repressive function of the *Meg3* expression or transcript, or conversely, with maternal *Meg3* expression facilitating CTCF recruitment or its stability of binding at the DMR, possibly through direct RNA-CTCF contacts [37, 38]. Importantly though, such a mechanism would be unique to the *Dlk1-Dio3* domain, as the *H19* lncRNA is not required for imprinted expression of the *Igf2* gene at the *Igf2-H19* domain [39].

High-resolution DNA-FISH and 4C-seq studies measure different aspects of chromatin organization. Whereas our FISH experiments describe inter-probe distances within a range of 1 μm measured in individual cells, the 4C-seq experiments are thought to detect in populations of cells the small subset of regions that co-localize at very short distance (several tens to maximum a few hundred nanometers; see for discussion, e.g., [40–42]). Joint consideration of both types of data can therefore provide complementary, and sometimes apparently paradoxical, insights into the mechanistic and functional organization of chromatin domains. At the *Dlk1-Dio3* domain, our 4C-seq studies identified the presence of the *Dlk1-Meg3* sub-TAD, yet our DNA-FISH studies revealed larger distances on the maternal chromosome between *Dlk1* and *Dio3* (Fig. 3 and Additional file 1: Figure S5). Instead of models based on a single source of data, suggesting that the entire maternal *Dlk1-Dio3* domain is either in a less compacted configuration (FISH) or sub-divided in two to three insulated sub-domains (4C-seq), the combination of both types of data suggests the presence of an intra-TAD 3D architecture where the *Dlk1-Meg3* sub-TAD physically loops away from the other sub-domains. This is supported by our three-way DNA-FISH studies where distances involving the central *Dlk1-Meg3* sub-TAD are longer than those between the flanking *Begain* and *Mirg-Dio3* sub-domains (Additional file 1: Figure S5d).

At the *Igf2-H19* domain, our DNA-FISH studies showed that average distances between loci on the parental chromosomes only differ for the *H19* DMR and a CTCF cluster located at its centromeric side. This appears at odds with our 4C-seq data for the same viewpoint that reveals a highly different pattern of chromatin loops uniquely formed on the maternal chromosome. Interestingly though, for viewpoints at the *H19* DMR and the 5′-located CTCF sites, we noticed a considerable enrichment of 4C-seq signal at the *Igf2* gene on the maternal allele as well (Fig. 2e, dotted arrow). The *H19* DMR therefore appears unable to impose the observed sub-TAD organization in all cells (or at all times), possibly due to its relatively low level of CTCF binding (Fig. 1a). This in turn may explain the reported incomplete maternal repression of *Igf2* [43]. We speculate that the array of four CTCF binding sites at the maternal *H19* DMR [4] hijacks 3D organization at the domain by acting as a ratchet for loops with the centromeric bi-allelic CTCF binding clusters, similarly as reported for the *Dxz4* region on the inactive X-chromosome [44]; however, this is insufficient to fully override the inherent paternal organization structured by the loops between the bi-allelic CTCF sites at *Igf2* and the same centromeric CTCF clusters. At the *Dlk1-Dio3* domain, a similar mechanism may structure the maternal *Dlk1-Meg3* sub-TAD. Here, the CTCF-bound maternal *Meg3* DMR interacts with two separate clusters of bi-allelic CTCF sites on the centromeric side of the sub-domain.

In conclusion, our study reveals how maternal-specific CTCF binding structures a further layer of sub-TAD organization that overrides the inherent paternal 3D chromatin organization and thus directly coincides with the implementation of the maternal transcriptional programs. Similarly as for the *H19* DMR—where epigenetic alterations that affect CTCF binding cause the growth-related imprinting disorders Beckwith-Wiedemann Syndrome (BWS) and Silver-Russell Syndrome (SRS) [2]—maternal CTCF binding at the *Meg3* DMR is evolutionarily conserved in humans [5]. Micro-deletions and gains of methylation within the human *MEG3* DMR have recently been linked to the developmental imprinting disorder Kagami-Ogata Syndrome (KOS14) [2, 45], indicating that our observations are relevant to humans as well.

## Methods

### ES cells, cell culture, and in vitro differentiation

Hybrid ESC lines BJ1 ((C57BL/6 J × JF1)F1) and JB1 ((JF1 × C57BL/6 J)F1) are both male and were derived in serum-free (2i) medium as part of a previous study [27]. These WT cells and the JB1- and BJ1-derived ESC lines with bi-allelic deletions comprising site 2 (this study), as well as our previously generated mono-parental ESC lines PR8 [46] and AK2 [47], were maintained without feeders on gelatin-coated dishes in serum-free ESGRO Complete PLUS medium (Millipore, with LIF and GSK3 inhibitor). The correct imprinted expression of the *Meg3* transcript in all cell lines was periodically confirmed, as well as the absence of mycoplasma contamination.

Differentiation of ESCs into cortical-identity neural progenitor cells (NPCs) was performed as described in detail before [27, 48]. Briefly, ES cells were plated on matrigel-coated dishes at a density of 3 × 10^5^ cells per 10-cm dish in serum-free ESGRO Complete PLUS medium, and after 24 h, the medium was changed to DDM (DMEM/F12 + GlutaMAX (ThermoFisher)), supplemented with 1× N2 (ThermoFisher), B27 (without vitamin A, ThermoFisher), 1 mM of sodium pyruvate, 500 μg/ml BSA, and 0.1 mM of 2-mercapto-ethanol for a total of 12 days. Cyclopamine (1 μM, Merck) was added from day 2 to day 10 of differentiation. Media was changed every 2 days. After 12 days of differentiation, NPCs were dissociated using StemPro Accutase and were used for high-throughput 4C studies. Part of the cells were replated on poly-lysine (Sigma)/laminin (Sigma) for expression studies, and cultured in 1:1 mixture of DDM and Neurobasal/N27 media (ThermoFisher, supplemented with 1× B27) and 2 mM GlutaMax) for further differentiation, till D21, or were re-plated onto coated coverslips to perform immunostainings and DNA-FISH studies 2 days later (D12+2).

### CRISPR-Cas9-mediated deletion of CTCF binding site 2 at *Meg3* intron1

The guide RNA (sgRNA) was designed using CRISPR Design tool (zlab.bio/guide-design-resources) and synthesized with *Bbs*I sticky ends: *Meg3* DMR CTCF site 2—GTTGCACATAGAGACCGCTAG. It was cloned into the pUC57-sgRNA expression vector [49] (a gift from Xingxu Huang; #51132, Addgene). The Cas9-VP12 vector [50] (a gift from Keith Joung; #72247, Addgene) was modified by adding T2A-GFP at the C-terminal end and electroporated with the sgRNA vector into JB1 and BJ1 hybrid ES cells using the Amaxa nucleofector procedure (Lonza). Twenty-four hours post-electroporation, GFP-positive cells were sorted by flow cytometry (FACS Aria, Becton Dickinson) and single cells were seeded onto 96-well plates. After 10–12 days of culture, individual colonies were picked and grown in 6-well plates. Genomic DNA was extracted, and the region around *Meg3* DMR CTCF site 2 was amplified (primers in Additional file 1: Table S2), followed by confirmation of the deletion by DNA sequencing (Additional file 1: Figure S6a).

### ChIP-qPCR, ChIP-seq, and data analysis

ChIP experiments were performed as previously described [51] with minor modifications. ESCs were fixed for 5 min in a 2% formaldehyde solution at room temperature. ChIP-seq samples were fragmented using a water bath sonicator (BioRuptor Plus, Diagenode). Ten micrograms of chromatin was immunoprecipitated with either 5 μg of CTCF antibody (07-729, Merck Millipore) or 4 μg of H3K27me3 antibody (17-622, Merck Millipore).

PR8, AK2, BJ1, and JB1 ChIP-qPCR samples were analyzed on a LightCycler 480 instrument (Roche Molecular Diagnostics) using the SsoAdvanced Universal SYBR Green Supermix (BioRad). Duplicate qPCR experiments were performed on technical replicates with recovery in each cell type expressed versus a corresponding input sample (primers in Additional file 1: Table S2).

Indexed ChIP-seq libraries were constructed using the Next Ultra Library Prep Kit for Illumina (New England Biolabs) using the application note “Low input ChIP-seq.” Multiplexed sequencing was done using 86-bp single-end reads on the Next-Seq 500 system (Illumina) at the I2BC Next Generation Sequencing Core Facility. Data were mapped to ENSEMBL Mouse assembly GRCm38 (mm10) using BWA with default parameters [52]. Reads for mono-parental PR8 and AK2 samples were further extended to 200 bp. After removal of duplicate, multiple aligning, and low-quality reads, densities in windows of 50 bp were calculated for combined technical replicates. Samples were normalized using quantile normalization after removal of regions with abnormal alignment in the input samples (either ≥ 3 IQR over median input signal or regions with no input signal at all). CTCF peaks in PR8 and AK2 ESCs were called if 4 consecutive 50 bp bins had a minimum value of 20 in at least 1 cell type, followed by extension of 1 bin left and right (Additional file 2). Differential CTCF peaks were called if the difference between peak values was ≥ 3-fold. We validated differential CTCF binding using ChIP-seq data from JB1 cells. After mapping to ENSEMBL Mouse assembly GRCm38 (mm10) using BWA, we identified known JF1 polymorphisms in the reads covering our identified CTCF peaks (ftp://molossinus.lab.nig.ac.jp/pub/msmdb/For_Seq_Analysis/list_of_variations/).

### RNA-seq and data analysis

Total RNA was extracted from AK2 and PR8 ESCs by lysing the cells on the culture dish with the addition of TRI reagent (Sigma-Aldrich). RNA samples were sequenced on the Illumina HiSeq-2000 system (Illumina) using the TruSeqTM SBS stranded mRNA sample kit (version 3). For both ES lines, RNA sequencing (2 × 100 bp) was performed in triplicate.

Paired-end Fastq files were mapped to ENSEMBL Mouse assembly GRCm38 (mm10) using STAR [53]. Transcript abundance was quantified using RSEM in TPM (transcript per million) for the 51,789 transcripts in EMSEMBL database version 89. Replicates showed high correlation (*R* ≥ 0.99) indicating good reproducibility and reliability. Samples were normalized against each other using quantile normalization, followed by averaging of triplicate samples (Additional file 3). Genes were considered significantly detected if the TPM value in either of the combined mono-parental PR8 or AK2 datasets was ≥ 5. Differential expression was called if the highest TPM value was ≥ 5, the fold difference between PR8 and AK2 TPM was ≥ 1.5, and the highest TPM value × the fold TPM difference was ≥ 50.

### RT-qPCR and allele-specific quantitation

Total RNA was extracted from hybrid ESCs and NPCs using the miRNEasy Kit (Qiagen) and DNaseI treatment (Qiagen). cDNA was synthesized from 5 μg of RNA using random hexamers and SuperScript III (ThermoFisher) reverse transcriptase. *Meg3* and *Dlk1* expression were quantified by RT-qPCR using SYBR Green I master mix (Roche) on a Lightcycler 480 instrument. Mean C_T_ values were normalized with the mean of two housekeeping genes (*Actb*, *Gapdh*) and the ΔΔCt method [54]. Primer sequences are in Additional file 1: Table S2.

The Taqman mutation detection assay was used for allele-specific quantitation (ThermoFisher). Expression was quantified using Taqman Genotyping Master Mix (ThermoFisher) and a Lightcycler 480 instrument. The levels were normalized to two housekeeping genes (*Actb*, *Gapdh*) amplified with SYBR Green Master Mix (Roche), as reported before [27].

### Reanalysis of Hi-C data

Raw data for ESCs and NPCs were obtained from GEO dataset GSE96107 [11]. HiC-Pro v2.9.0, using Bowtie2 v2.3.0, was used to map the raw data to mouse reference genome mm10 and to process the aligned reads, with default settings to remove duplicates, assign reads to *Dpn*II restriction fragments, and filter for valid interactions [55, 56]. Binned interaction matrices were generated at 10-kb resolution from the valid interactions and were normalized using the Iterative Correction and Eigenvector decomposition method (ICE) implemented in HiC-Pro. TAD borders were called using TADtool [24], with window size 500 kb and insulation index cutoff value 21.75, resulting in a high degree of genome-wide overlap with TAD borders as reported by Bonev et al. [11].

### 4C-seq and data analysis

Chromatin fixation, cell lysis, and 4C library preparation were done as previously described [57] using 15 million cells per experiment, *DpnII* (New England Biolabs) as the primary restriction enzyme and *NlaIII* (New England Biolabs) as the secondary restriction enzyme. For 4C-seq library preparation, 800 ng of 4C library was amplified using 16 individual PCR reactions with inverse primers including the Solexa or TruSeq adapter sequences (primers in Additional file 1: Table S2). Illumina sequencing was done on samples containing PCR amplified material of up to ten viewpoints using 100 bp or 86 bp single-end reads on the Illumina Hi-Seq 2500 or Next-Seq 500 systems at the iGE3 Genomics Platform of the University of Geneva (Switzerland) or the I2BC Next Generation Sequencing Core Facility. 4C-seq datasets were mapped and translated into restriction fragments using the 4C-seq pipeline of the BBCF HTSstation [58], according to ENSEMBL Mouse assembly GRCm38 (mm10). For visualization of 4C-seq patterns, smoothed 4C-seq data (11 fragments) were normalized to the signal within the region covering the 5 TADs surrounding the viewpoint, as described previously [59] (see also Additional file 1: Figure S2c, d). Regions for normalization were as follows: *Igf2-H19* locus—chr7:141,530,000-143,520,000; *Dlk1-Dio3* locus—chr12:105,970,000-110,860,000. Ratios between smoothed 4C-seq patterns were calculated using the BioScript library of the BBCF HTS station [58]. Distributions of 4C-seq signal were calculated using a previously described approach [59], with unprocessed 4C-seq data normalized within the previously mentioned 5 TADs and signal within each sub-domain expressed per Mb. Significant differences between 4C-seq signal on the maternal and paternal chromosomes at the *Igf2-H19* locus (adjusted *p* value < 0.01) were detected with the FourCSeq package, using the smoothed 4C-seq replicate profiles (window size = 5) of the *H19* DMR viewpoint and the following parameters: getZscores function with minCount = 10 and minDist = 20 kb [25]. Significant differences between individual sub-TADs or between sub-TADs on the maternal and paternal chromosomes were calculated using a previously applied approach [51, 60], by determining the fraction of fragments with increased maternal versus paternal signal in the sub-domains versus the remainder of the TAD or between sub-domains, followed by a *G* test of independence.

### DNA methylation analysis

DNA methylation was analyzed by digestion of genomic DNA samples with a methylation-sensitive restriction endonuclease, followed by qPCR amplification and Sanger sequencing of the PCR products [27]. Briefly, 1 μg of genomic DNA was fragmented with *Eco*RI, after which half of the reaction was further digested with the methylation-sensitive enzyme *Aci*I. One nanogram of both the *Aci*I-digested DNA and of the non-digested (*Eco*RI only) DNA was used for qPCR. Quantitative values were obtained using the standard curve method [61] with normalization against two regions without *Aci*I sites (*Col1a2* and *Col9a2*). The *ActB* gene (unmethylated *Aci*I site) and IAP retrotransposons (methylated *Aci*I sites) were included as controls. Primer sequences are in Additional file 1: Table S2.

### Probes for 3D DNA-FISH and RNA-FISH

Fosmid and BAC probes were directly labeled by nick translation (Abbott molecular, ref. 07 J00-001) with Cy3-dUTP or Cy5-dUTP (GE Healthcare). Details on fosmids and BACs are provided in Additional file 1: Table S3. Per coverslip, 0.1 μg of nick-translation product was precipitated in the presence of 10 μg of salmon sperm and 5 μg of Cot-1 DNA and resuspended in 10 μl of hybridization buffer (50% formamide, 2X SSC, 10% dextran sulfate, 1 mg/ml BSA, 20 mM VRC; pH 7.0) and denatured for 7 min at 75 °C. Competition was done for 30 min at 37 °C (this step was not applied for RNA-FISH) before overnight hybridization of the cells. For RNA-FISH, 24 oligonucleotides of 54–60 nt in length were designed to *Meg3* (see [30] for probe sequences) and synthesized with 28-nt “FLAP sequences” and were hybridized by secondary fluorescent probes as described [62].

### 3D DNA-FISH

3D DNA-FISH was carried out on ESCs adhered to 0.1% gelatin-coated coverslips as previously described [27]. Briefly, cells were fixed in (3% paraformaldehyde, 1×PBS; pH 7.4) for 10 min at RT and permeabilized for 7 min with (0.5% Triton X-100, 1×PBS; pH 7.4) on ice, and denatured at 80 °C in (50% formamide, 2×SSC; pH 7.0) for 30 min. Cells were rinsed in ice-cold 2×SSC; pH 7.0, and hybridized with probes overnight at 42 °C (coverslips were sealed onto slides with rubber cement). The next day, cells were washed 3 times in (50% formamide, 2×SSC; pH 7.2) at 42 °C, and 3 times in 2×SSC; pH 7.0 at 42 °C for 5 min each. Finally, coverslips were stained with DAPI and mounted using Vectashield antifade mounting medium (VectorLabs, H-1000).

### DNA-RNA FISH with MS2 oligo probes

Fixation of hybrid BJ1 ESCs containing 64 copies of MS2 repeats into exon-10 of the *Meg3* lncRNA was done as previously described [30]. Following fixation, cells were incubated twice for 5 min with DEPC-treated 1×PBS; pH 7.4 at RT and dehydrated in 80%, 95%, and 100% ethanol, for 3 min each respectively, and were air dried. Then, cells were rehydrated in (20% formamide, 2×SSC, 0.01% Tween 20; pH 7.0) for 5 min at 37 °C. An MS2-multi-oligonucleotide probe was mixed in 10 μl of hybridization buffer (20% formamide, 2×SSC, 10% dextran sulfate, 50 mM sodium phosphate, 2 mM VRC; pH 7.0) that was pre-warmed at 37 °C. Probes were denatured 1 min at 80 °C, and cells were hybridized with probes for 2 h at 37 °C in a dark and humid chamber. Coverslips were washed 3 times with (20% formamide, 2×SSC, 0.01% Tween 20; pH 7.0) for 5 min at 37 °C, and once briefly in DEPC-treated 1×PBS; pH 7.4. Cells were post-fixed in 4% paraformaldehyde, 1×PBS; pH 7.4 for 10 min at RT and rinsed 3 times in DEPC-treated 1×PBS; pH 7.4 for 5 min. Cells were incubated in 2×SSC; pH 7.0 for 5 min at 40 °C, following denaturation in (70% formamide, 2×SSC, 50 mM sodium phosphate buffer; pH 7.0) for 3 min at 73 °C, and then in (50% formamide, 2×SSC, 50 mM sodium phosphate buffer; pH 7.0) for 1 min at 73 °C. Cells were hybridized with prepared fosmid probes overnight at 37 °C (coverslips were sealed onto slides with rubber cement). The next day, cells were washed 3 times in (50% formamide, 2×SSC, 0.01% Tween 20; pH 7.2) at 42 °C for 5 min each, and 3 times in (2×SSC; pH 7.0) at 42 °C for 5 min each. Finally, coverslips were stained with DAPI and mounted using Vectashield antifade mounting medium.

### Immunofluorescence

Immunofluorescence staining was performed as described [48]. Primary antibodies were as follows: anti-Nestin (839801, Biolegend; 1:1000 dilution) and anti-Tubb3 (801201, Biolegend; 1:1000 dilution). Secondary antibodies were as follows: goat anti-mouse Alexa Fluor 488 (A-11011, Life Technologies; 1:1000 dilution) and goat anti-rabbit Alexa Fluor 594 (A-11012, Life Technologies; 1:1000 dilution).

### Confocal microscopy and data analysis

Three-dimensional images were acquired with a Zeiss LSM780 laser scanning confocal microscope (Zeiss), using a 63×NA 1.4 Plan-Apochromat oil immersion objective. Optical sections separated by 0.4 μm steps were collected in the Z direction. Stacks were analyzed using Imaris software (Bitplane, Switzerland). FISH signals were segmented in 3D, and their centers of mass were defined. For double FISH experiments, the distances between closest neighbor’s centers of mass were calculated. Only FISH fluorescence signals within DAPI 3D-segmented object were considered for the analysis.

Measured distances between BAC probe FISH signals were normalized to the genomic distances and not to the radius of individual nuclei, because no differences in cell radius were observed between the three cell lines (PR8, AK2, and BJ1) (not shown). Significance of differences between distances from combined repeated experiments of DNA probes was calculated using the two-tailed unpaired Mann-Whitney *t* test.

## Supplementary information

**Additional file 1.** Supplementary Figures and Supplementary Tables.

**Additional file 2.** CTCF binding peaks in the *Dlk1-Dio3* TAD in mono-parental ESCs (ChIP-seq).

**Additional file 3.** RNA transcript levels of genes in the *Igf2-H19* and *Dlk1-Dio3* TADs in mono-parental ESCs (RNA-seq).

**Additional file 4.** Review history.

## Data Availability

Published Hi-C sequences for ESCs and NPCs were obtained from the NCBI GEO dataset GSE96107 [63]. Unprocessed ChIP-seq, RNA-seq, and 4C-seq sequences generated in this study are available from the European Nucleotide Archive (EMBL-EBI ENA) repository under accession number PRJEB28762 [64]. Processed 4C-seq interaction patterns are available from the Mendeley Data repository [65]. Raw microscopy data is available from the Figshare repository [66].
