# Supplementary material for: CTCF modulates allele-specific sub-TAD organization and imprinted gene activity at the mouse Dlk1-Dio3 and Igf2-H19 domains
Source: Genome Biol. 2019 Dec 12;20:272. doi: 10.1186/s13059-019-1896-8 (PMC6909504; doi:10.1186/s13059-019-1896-8)
Supplement: Supplementary file 1 — Additional file 1. Supplementary Figures and Supplementary Tables. [file 13059_2019_1896_MOESM1_ESM.pdf]

Additional file 1

CTCF modulates allele-specific sub-TAD organisation and imprinted gene activity at the mouse *Dlk1-Dio3* and *Igf2-H19* domains

David Llères, Benoît Moindrot, Rakesh Pathak, Vincent Piras, Mélody Matelot, Benoît Pignard, Alice Marchand, Mallory Poncelet, Aurélien Perrin, Virgile Tellier, Robert Feil and Daan Noordermeer

Table of content:

Figure S1. Multiple instances of bi-allelic CTCF binding accompany maternal allele-specific CTCF binding at the *Meg3* and *H19* DMRs 2

Figure S2. DNA interactions at paternally imprinted gene domains are confined within the same TADs on both the parental chromosomes 4

Figure S3. The *Igf2-H19* domain adopts an allele-specific sub-TAD organisation that is anchored by bi-allelic and allele-specific CTCF sites 6

Figure S4. A maternal *Dlk1-Meg3* sub-TAD is structured by bi-allelic and allele-specific CTCF sites 8

Figure S5. The *Dlk1-Dio3* domain is organised into allele-specific sub-TADs that coincide with different allelic intra-TAD distances 10

Figure S6. CTCF binding at site 2 in the *Meg3* DMR is required for the structure of the *Dlk1-Meg3* sub-TAD 12

Figure S7. CTCF binding at site 2 in the *Meg3* DMR is required for correct imprinted activation of *Dlk1* 14

Figure S8. Developmental *Dlk1* activation on the maternal chromosome coincides with a mostly stable intra-TAD organisation 16

Figure S9. Comparison of 3D chromatin organization of the *Igf2-H19* domain to previously published data 18

Table S1. Significance of 4C-seq signal in sub-TADs 19

Table S2. Primers 21

Table S3. FISH Probes 24

Supplemental references 25

FIGURE S1

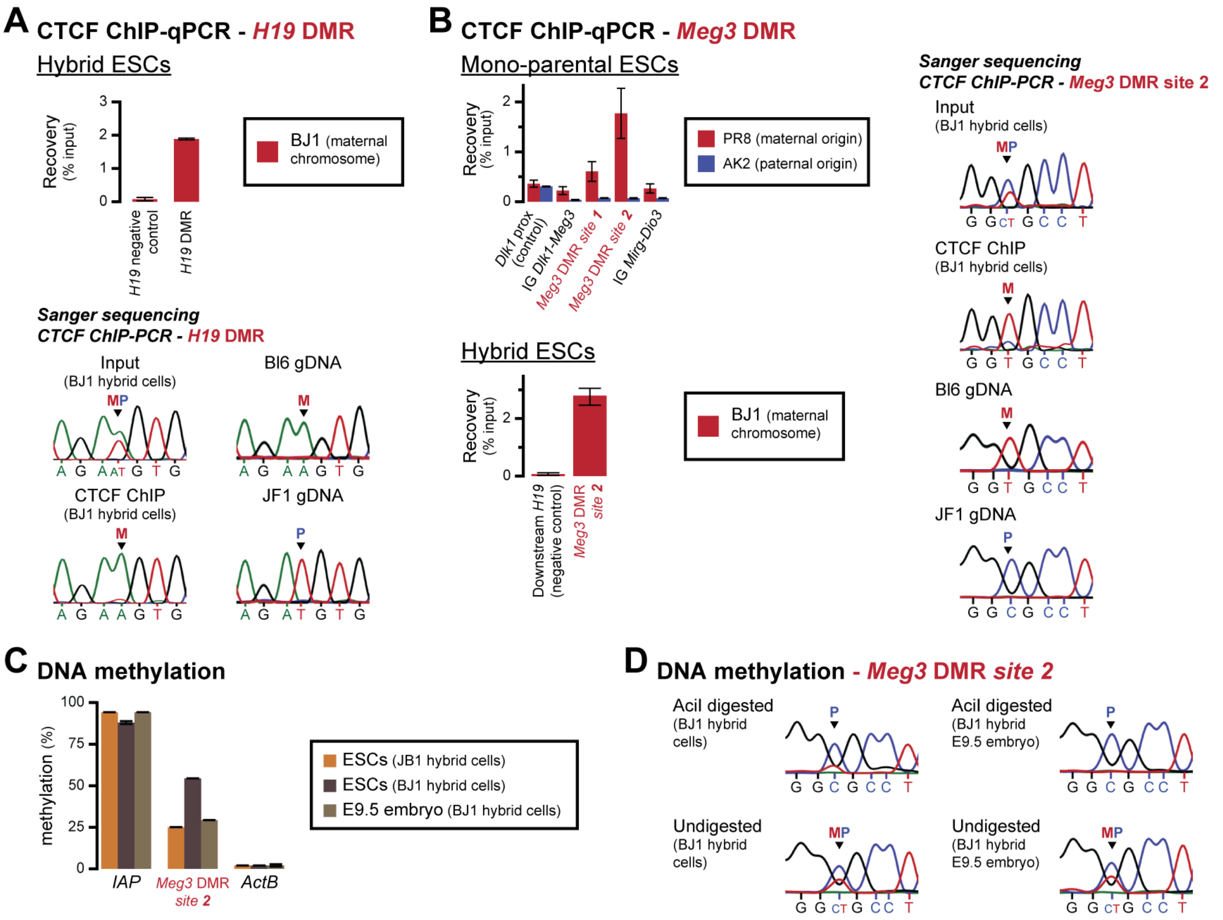

A CTCF ChIP-qPCR - H19 DMR
Hybrid ESCs
Recovery (% input)
H19 negative control
H19 DMR
BJ1 (maternal chromosome)
Sanger sequencing
CTCF ChIP-PCR - H19 DMR
Input (BJ1 hybrid cells)
Bl6 gDNA
CTCF ChIP (BJ1 hybrid cells)
JF1 gDNA
B CTCF ChIP-qPCR - Meg3 DMR
Mono-parental ESCs
Dlk1 prox (control)
IG Dlk1-Meg3
Meg3 DMR site 1
Meg3 DMR site 2
IG Mirg-Dio3
PR8 (maternal origin)
AK2 (paternal origin)
Hybrid ESCs
Downstream H19 (negative control)
BJ1 (maternal chromosome)
Sanger sequencing
CTCF ChIP-PCR - Meg3 DMR site 2
C DNA methylation
methylation (%)
IAP
ActB
ESCs (JB1 hybrid cells)
ESCs (BJ1 hybrid cells)
E9.5 embryo (BJ1 hybrid cells)
D DNA methylation - Meg3 DMR site 2
AciI digested (BJ1 hybrid cells)
AciI digested (BJ1 hybrid E9.5 embryo)
Undigested (BJ1 hybrid cells)
Undigested (BJ1 hybrid E9.5 embryo)

Figure S1. Multiple instances of bi-allelic CTCF binding accompany maternal allele-specific CTCF binding at the *Meg3* and *H19* DMRs

A. ChIP-qPCR (top) and ChIP-PCR followed by Sanger sequencing (bottom) confirms maternal allele-specific CTCF binding at the *H19* DMR in hybrid ESCs. Error bars indicate Standard Error of the Mean (SEM) from 2 replicates (n = 2).

B. Top left: ChIP-qPCR validation of maternal allele-specific CTCF binding at the *Dlk1-Dio3* locus in mono-parental PR8 and AK2 ESCs. Error bars indicate SEM (n = 2).

Bottom left: ChIP-qPCR validation of maternal allele-specific CTCF binding at site 2 in the *Meg3* DMR in hybrid ESCs. Error bars indicate SEM (n = 2).

Right: ChIP-PCR followed by Sanger sequencing (bottom) confirms maternal allele-specific CTCF binding at site 2 in the *Meg3* DMR in hybrid ESCs.

C. DNA methylation levels as determined by digestion of genomic DNA from hybrid ESCs and E9.5 hybrid embryos with *Aci*I, an endonuclease that cuts non-methylated DNA only. Values are expressed as percentage of non-digested DNA. Positive control: *IAP* transposable elements (high levels of methylation) and negative control: *ActB* promoter (low levels of methylation). Methylation of CTCF site 2 in the *Meg3* DMR in ESCs is in a similar range as in E9.5 embryo. Error bars indicate SEM (n = 2).

D. Confirmation of maternal-specific DNA methylation in hybrid ESCs and E9.5 embryos by Sanger sequencing of genomic DNA with (top) and without (bottom) methylation-sensitive *Aci*I digestion. Parental origin of the SNP that distinguishes the maternal and paternal alleles is indicated. See also Fig. 1D.

FIGURE S2

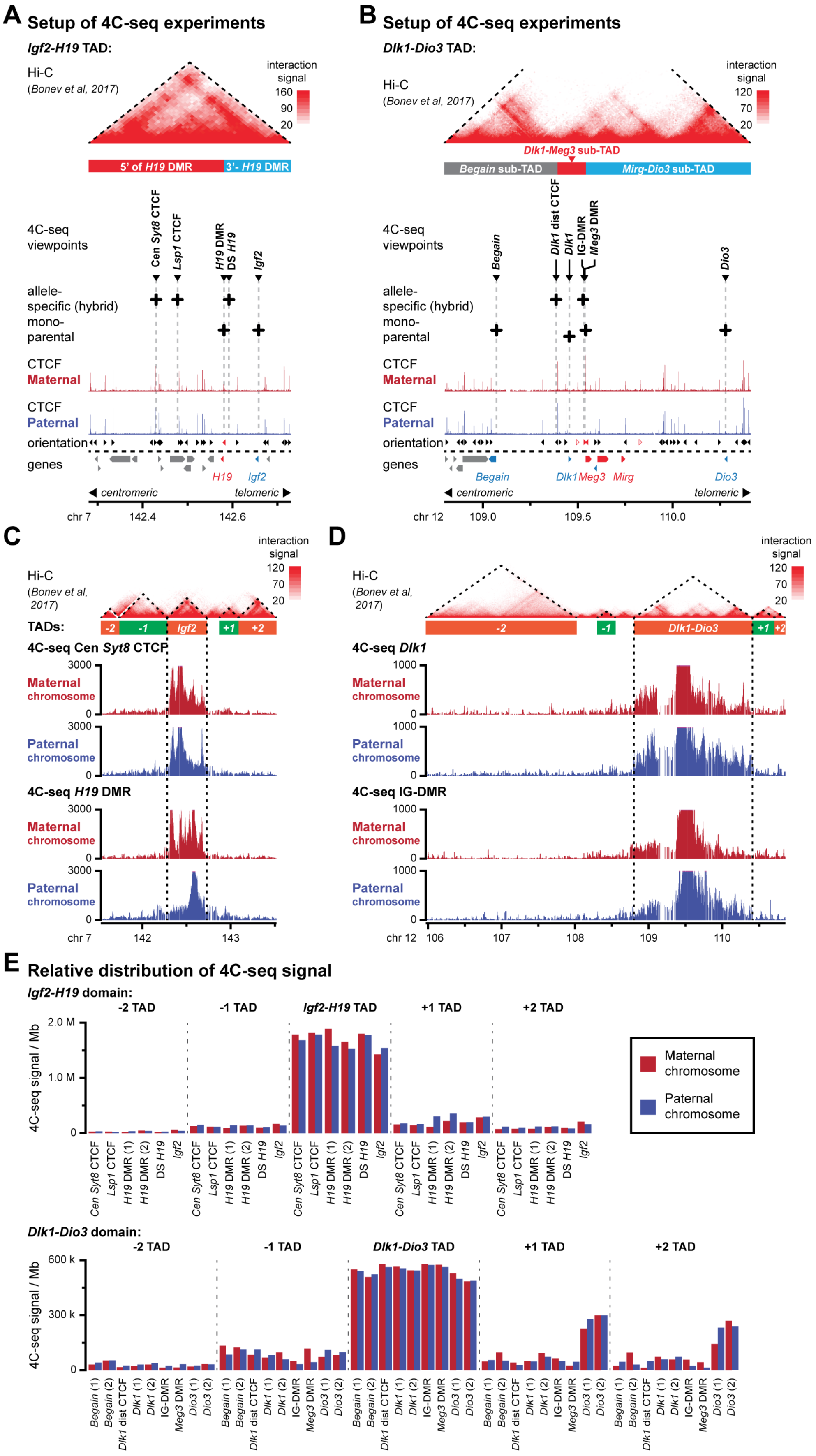
A Setup of 4C-seq experiments
Igf2-H19 TAD:
Hi-C
(Bonev et al, 2017)
interaction signal
160
90
20
5' of H19 DMR
3'- H19 DMR
4C-seq viewpoints
Cen Syt8 CTCF
Lsp1 CTCF
H19 DMR
DS H19
Igf2
allele-specific (hybrid)
mono-parental
CTCF Maternal
CTCF Paternal
orientation
genes
H19
Igf2
centromeric
telomeric
chr 7
142.4
142.6
B Setup of 4C-seq experiments
Dlk1-Dio3 TAD:
Hi-C
(Bonev et al, 2017)
interaction signal
120
70
20
Dlk1-Meg3 sub-TAD
Begain sub-TAD
Mirg-Dio3 sub-TAD
4C-seq viewpoints
Begain
Dlk1 dist CTCF
Dlk1
IG-DMR
Meg3 DMR
Dio3
allele-specific (hybrid)
mono-parental
CTCF Maternal
CTCF Paternal
orientation
genes
Begain
Dlk1
Meg3
Mirg
Dio3
centromeric
telomeric
chr 12
109.0
109.5
110.0
C
Hi-C
(Bonev et al, 2017)
interaction signal
120
70
20
TADs:
-2
-1
Igf2
+1
+2
4C-seq Cen Syt8 CTCF
Maternal chromosome
Paternal chromosome
3000
0
4C-seq H19 DMR
chr 7
142
143
D
Hi-C
(Bonev et al, 2017)
interaction signal
120
70
20
-2
-1
Dlk1-Dio3
+1
+2
4C-seq Dlk1
Maternal chromosome
Paternal chromosome
1000
0
4C-seq IG-DMR
chr 12
106
107
108
109
110
E Relative distribution of 4C-seq signal
Igf2-H19 domain:
-2 TAD
-1 TAD
Igf2-H19 TAD
+1 TAD
+2 TAD
4C-seq signal / Mb
2.0 M
1.0 M
0
Cen Syt8 CTCF
Lsp1 CTCF
H19 DMR (1)
H19 DMR (2)
DS H19
Igf2
Maternal chromosome
Paternal chromosome
Dlk1-Dio3 domain:
-2 TAD
-1 TAD
Dlk1-Dio3 TAD
+1 TAD
+2 TAD
4C-seq signal / Mb
600 k
300 k
0
Begain (1)
Begain (2)
Dlk1 dist CTCF
Dlk1 (1)
Dlk1 (2)
IG-DMR
Meg3 DMR
Dio3 (1)
Dio3 (2)

Figure S2. DNA interactions at paternally imprinted gene domains are confined within the same TADs on both the parental chromosomes

A. Set-up of 4C-seq experiments at the paternally imprinted *Igf2-H19* domain and its surrounding TAD. The positions of viewpoints in hybrid cells (allele-specific) or mono-parental cells are indicated within the overarching TAD. Allele-specific CTCF signal is indicated below. Non-allelic Hi-C signal and the position of the maternal sub-TADs are indicated above. The orientation of CTCF sites, genes and centromeric and telomeric orientation are indicated below with colours indicating allele-specificity. Non-allelic Hi-C data are from reference [1].

B. Set-up of 4C-seq experiments at the paternally imprinted *Dlk1-Dio3* domain and its surrounding TAD.

C. 4C-seq signal for two viewpoints in the *Igf2-H19* domain on the maternal (red) and paternal (blue) chromosome in mono-parental and hybrid ESCs. Signal is indicated in the 5 TADs surrounding the viewpoint (orange-green blocks), with reanalysed Hi-C signal visualized above. Allele-specific CTCF signal (ChIP-seq on mono-parental ESCs) is provided below. Non-allelic Hi-C data are from reference [1].

D. 4C-seq signal for two viewpoints in the *Dlk1-Dio3* domain on the maternal (red) and paternal (blue) chromosome in mono-parental and hybrid ESCs.

E. Relative distribution of 4C-seq signal for indicated viewpoints in the 5 TADs surrounding the two imprinted domains.

FIGURE S3

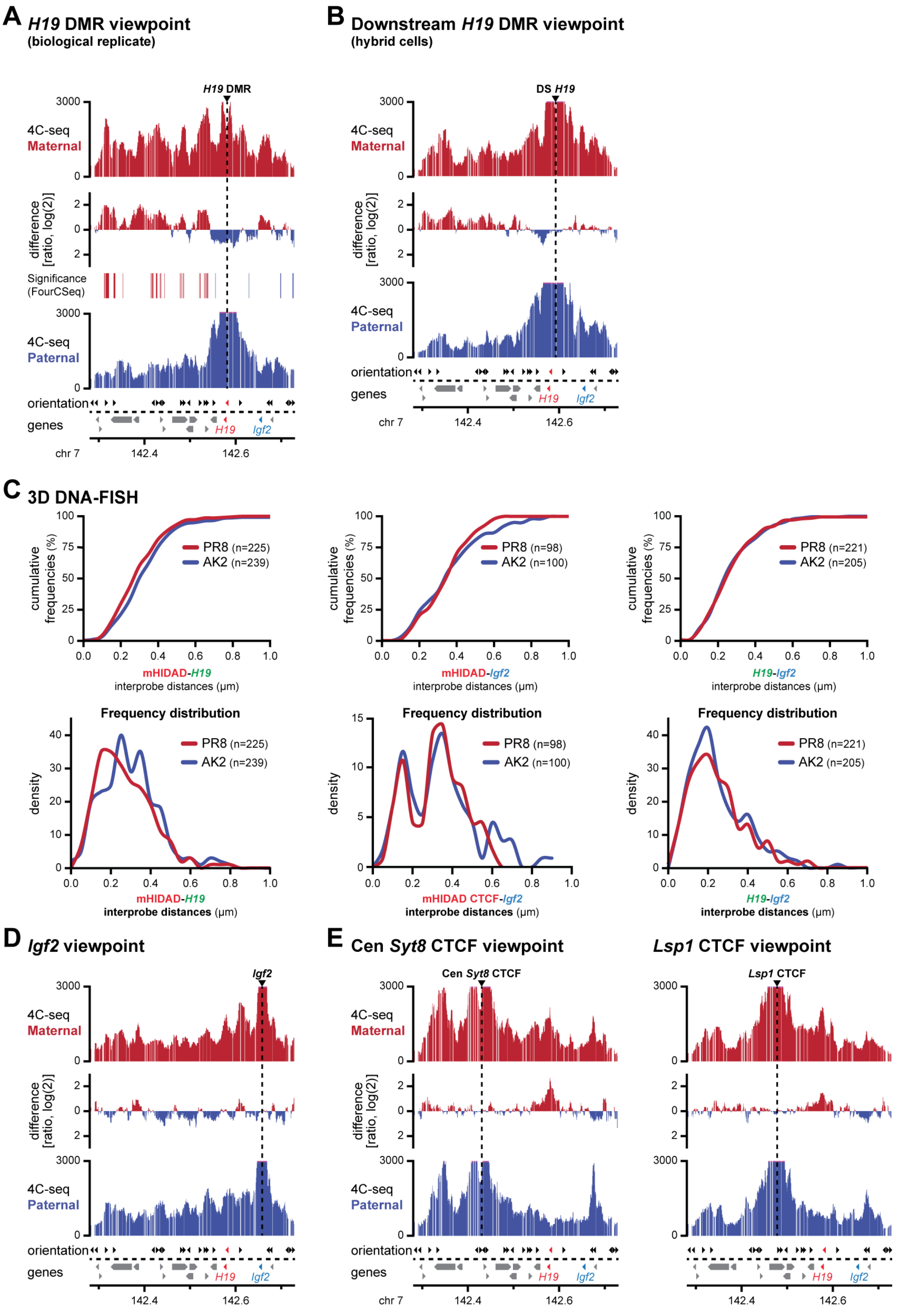
A H19 DMR viewpoint
(biological replicate)
B Downstream H19 DMR viewpoint
(hybrid cells)
H19 DMR
DS H19
4C-seq
Maternal
Paternal
difference
[ratio, log(2)]
Significance
(FourCSeq)
orientation
genes
H19
Igf2
chr 7
142.4
142.6
C 3D DNA-FISH
cumulative
frequencies (%)
PR8 (n=225)
AK2 (n=239)
PR8 (n=98)
AK2 (n=100)
PR8 (n=221)
AK2 (n=205)
mHIDAD-H19
mHIDAD-Igf2
H19-Igf2
interprobe distances (µm)
Frequency distribution
density
mHIDAD CTCF-Igf2
D Igf2 viewpoint
E Cen Syt8 CTCF viewpoint
Lsp1 CTCF viewpoint
Cen Syt8 CTCF
Lsp1 CTCF

Figure S3. The *Igf2-H19* domain adopts an allele-specific sub-TAD organisation that is anchored by bi-allelic and allele-specific CTCF sites

A. 4C-seq signal from a biological replicate for the *H19* DMR viewpoint on the maternal (red) and paternal (blue) chromosomes in mono-parental ESCs. The ratio of interactions is provided between the patterns, with significant differences in both replicates, as determined by FourCSeq [2], indicated below.

B. 4C-seq signal for a viewpoint located 10 kb telomeric from the *H19* DMR in hybrid ESCs.

C. Distance distribution and cumulative distance frequencies between indicated fosmid probes in mono-parental ESCs.

D. 4C-seq signal for the *Igf2* viewpoint in mono-parental ESCs.

E. 4C-seq signal for two bi-allelic CTCF peaks (left: CTCF cluster centromeric from the *Syt8* gene and right: CTCF cluster in the *Lsp1* gene) in hybrid ESCs.

FIGURE S4

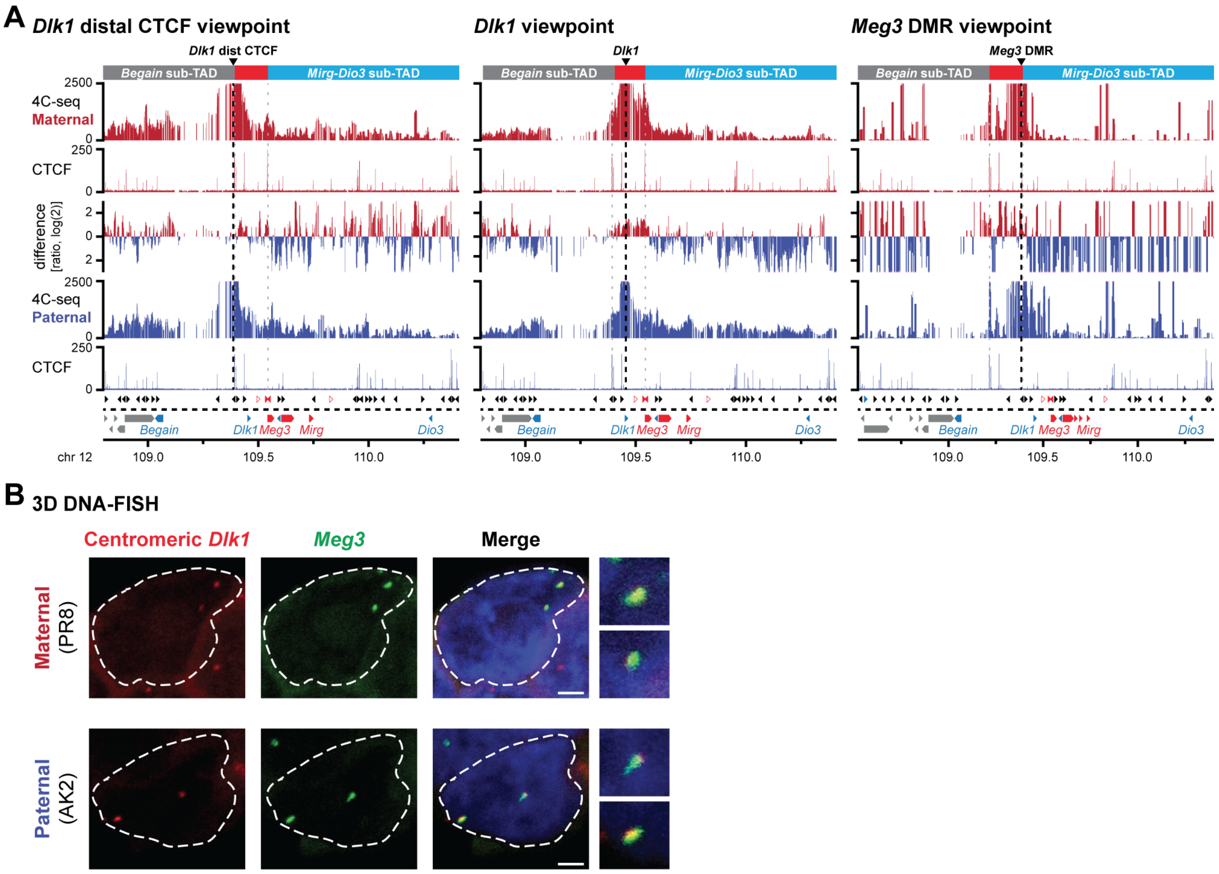
A
Dlk1 distal CTCF viewpoint
Dlk1 viewpoint
Meg3 DMR viewpoint
Dlk1 dist CTCF
Dlk1
Meg3 DMR
Begain sub-TAD
Mirg-Dio3 sub-TAD
4C-seq
Maternal
CTCF
difference
[ratio, log(2)]
Paternal
2500
250
0
2
Begain
Dlk1
Meg3
Mirg
Dio3
chr 12
109.0
109.5
110.0
B
3D DNA-FISH
Centromeric Dlk1
Meg3
Merge
Maternal
(PR8)
Paternal
(AK2)

<u>Figure S4. A maternal *Dlk1-Meg3* sub-TAD is structured by bi-allelic and allele-specific CTCF sites</u>

A. 4C-seq signal for indicated viewpoints in the *Dlk1-Meg3* sub-TAD on the maternal (red) and paternal (blue) alleles in the 1.6-Mb *Dlk1-Dio3* TAD. The ratio of interactions is provided in-between the patterns. Allele-specific CTCF signal is indicated below each 4C pattern. 4C-seq viewpoints and sub-TADs are indicated above. The orientation of CTCF sites and genes are indicated below with colours indicating allele-specificity.

B. Representative examples of DNA-FISH using fosmid probes in mono-parental ESCs. Scale bar, 2 µm. See also Fig. 3C.

FIGURE S5

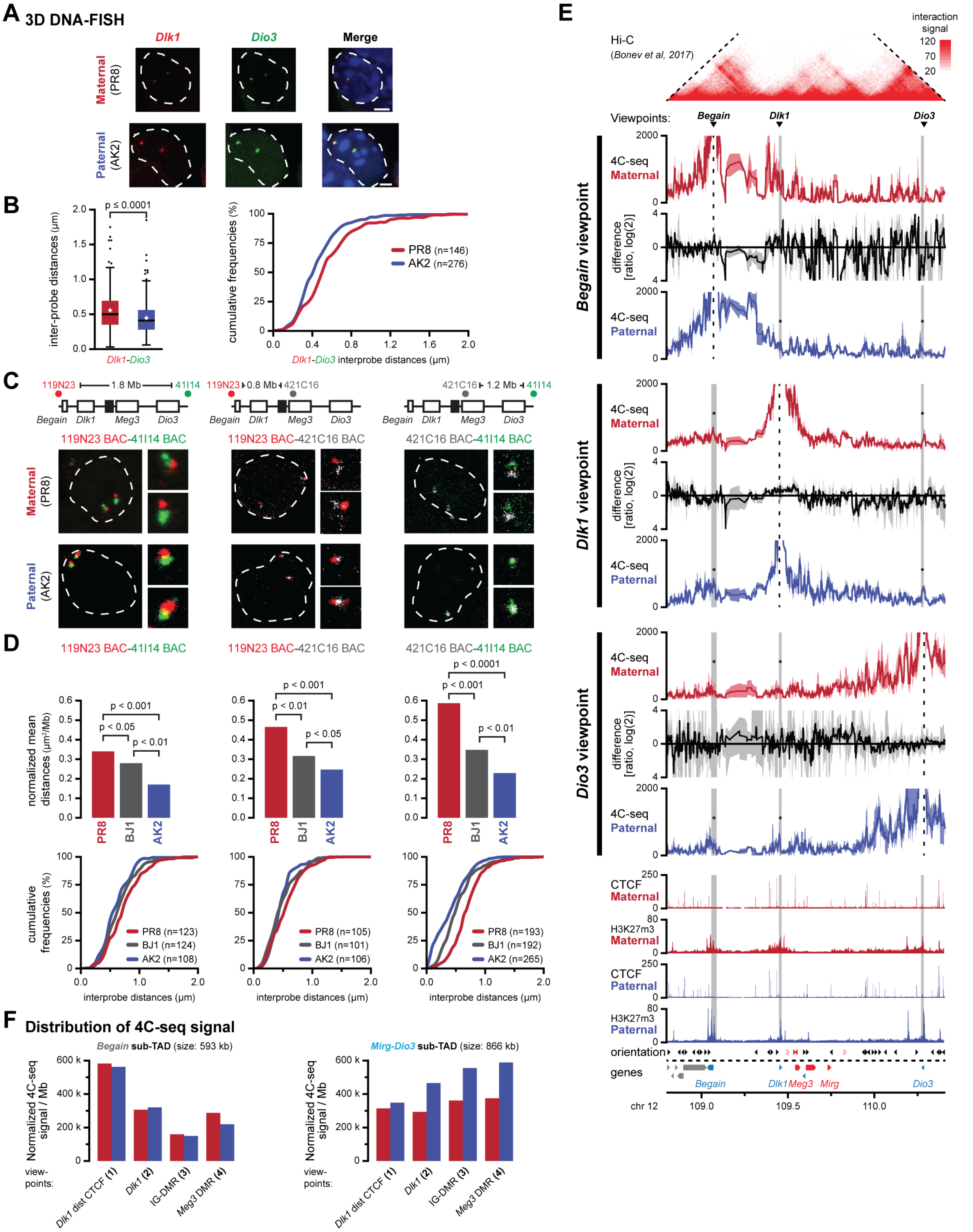
A 3D DNA-FISH
Dlk1
Dio3
Merge
Maternal (PR8)
Paternal (AK2)
B
p ≤ 0.0001
inter-probe distances (µm)
Dlk1-Dio3
cumulative frequencies (%)
PR8 (n=146)
AK2 (n=276)
Dlk1-Dio3 interprobe distances (µm)
C
119N23
1.8 Mb
41I14
0.8 Mb
421C16
1.2 Mb
Begain
Dlk1
Meg3
Dio3
119N23 BAC-41I14 BAC
119N23 BAC-421C16 BAC
421C16 BAC-41I14 BAC
Maternal (PR8)
Paternal (AK2)
D
119N23 BAC-41I14 BAC
119N23 BAC-421C16 BAC
421C16 BAC-41I14 BAC
normalized mean distances (µm²/Mb)
p < 0.001
p < 0.05
p < 0.01
p < 0.0001
PR8
BJ1
AK2
cumulative frequencies (%)
PR8 (n=123)
BJ1 (n=124)
AK2 (n=108)
PR8 (n=105)
BJ1 (n=101)
AK2 (n=106)
PR8 (n=193)
BJ1 (n=192)
AK2 (n=265)
interprobe distances (µm)
E
Hi-C
(Bonev et al, 2017)
interaction signal
Viewpoints:
Begain
Dlk1
Dio3
Begain viewpoint
Dlk1 viewpoint
Dio3 viewpoint
4C-seq Maternal
difference [ratio, log(2)]
4C-seq Paternal
CTCF Maternal
H3K27m3 Maternal
CTCF Paternal
H3K27m3 Paternal
orientation
genes
Begain
Dlk1
Meg3
Mirg
Dio3
chr 12
109.0
109.5
110.0
F Distribution of 4C-seq signal
Begain sub-TAD (size: 593 kb)
Mirg-Dio3 sub-TAD (size: 866 kb)
Normalized 4C-seq signal / Mb
view-points:
Dlk1 dist CTCF (1)
Dlk1 (2)
IG-DMR (3)
Meg3 DMR (4)

<u>Figure S5. The *Dlk1-Dio3* domain is organised into allele-specific sub-TADs that coincide with different allelic intra-TAD distances</u>

A. Examples of 3D DNA-FISH with fosmid probes in the *Dlk1-Dio3* TAD. Images show representative cells in mono-parental ESCs. Scale bars, 2 µm.

B. Distance measurements in mono-parental cells confirm the increased separation between *Dlk1* and *Dio3* on the maternal chromosome.

C. Representative examples of DNA-FISH using three BAC probes in mono-parental and hybrid ESCs. Scale bars, 1 µm. The schematic location of the BAC probes is indicated above.

D. Distance measurements in mono-parental and hybrid cells confirm the increased separation between all combinations of BAC probes on the maternal chromosome.

E. 4C-seq line-graphs for the inactive, H3K27me3-marked protein-coding genes at the *Dlk1-Dio3* locus. Lines indicate the average 4C-seq signal from 2-3 replicates/viewpoint, with surface showing maximum and minimum values. The ratio of interactions between replicate experiments is provided between the patterns, with the surface showing maximum difference between samples. The *Dlk1-Dio3* TAD (non-allele specific Hi-C signal; [1]) and the 4C-seq viewpoints are indicated above. Allele-specific CTCF and H3K27me3 signal (ChIP-seq), the orientation of CTCF sites and genes are indicated below. A moderate enrichment of interactions at or near the H3K27me3-marked promoters of the imprinted protein-coding genes within the locus may be observed (asterisks), yet with little difference between the maternal and paternal alleles.

F. Distribution of 4C-seq signal for indicated viewpoints in the *Begain* sub-TAD (left) and the *Mirg-Dio3* sub-TAD (right). Whereas interactions in the *Begain* sub-TAD are largely invariant between the parental chromosomes, in the *Mirg-Dio3* sub-TAD they are consistently enriched on the paternal chromosome.

FIGURE S6

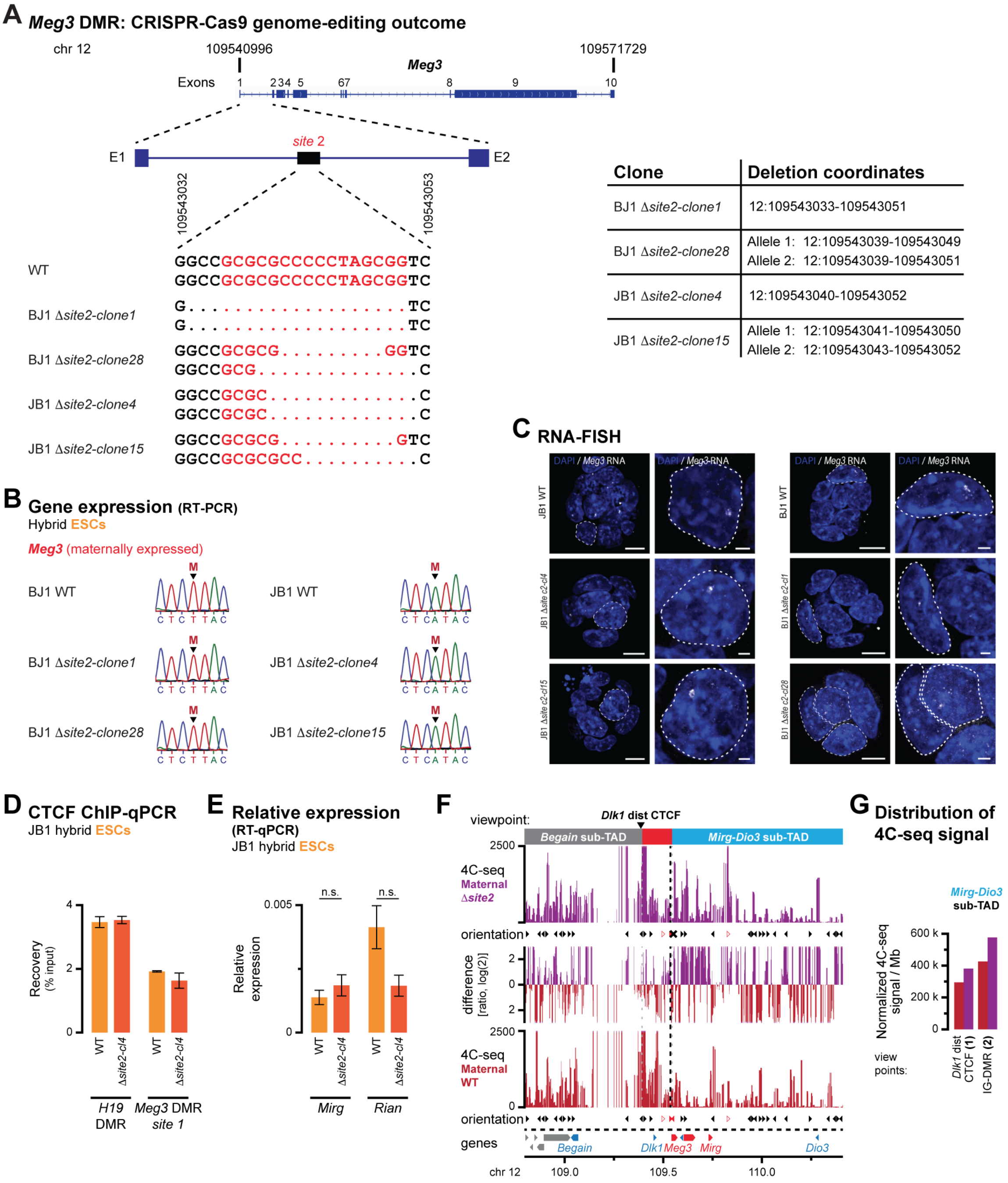

<u>**Figure S6. CTCF binding at site 2 in the *Meg3* DMR is required for the structure of the *Dlk1-Meg3* sub-TAD**</u>

A. Location of CTCF binding site 2 in the *Meg3* gene (top) and genotyping of the CTCF binding site deletions in the hybrid BJ1 and JB1 clones generated in this study (bottom). Coordinates of deletions (mm10) are provided in the panel on the right.

B. Sanger sequencing of RT-PCR products confirms continued maternal allele-specific *Meg3* expression in JB1- and BJ1-derived hybrid ESC lines carrying a deletion in CTCF binding site 2 in the *Meg3* DMR. The parental origin of the SNP that distinguishes the maternal and paternal alleles is indicated.

C. Single-molecule inexpensive-FISH (smiFISH) detection of *Meg3* RNA (white) in JB1- and BJ1-derived hybrid ESC lines carrying a deletion in CTCF binding site 2 in the *Meg3* DMR and their WT counterparts. The zoom-in of each cell shows a single bright RNA accumulation, indicating the active transcription site, and additional small spots elsewhere in the nucleoplasm, likely corresponding to individual *Meg3* mRNA molecules.

D. ChIP-qPCR validation of CTCF binding at site 1 in the *Meg3* DMR in hybrid ESCs. Error bars indicate SEM from 2 replicates.

E. Expression levels of *Mirg* and *Rian* non-coding RNAs in ESCs with a deleted CTCF site 2 in the *Meg3* DMR or their WT counterparts. Error bars indicate SEM (n = 2), with significance of difference determined using a two-sided unpaired t-test (n.s.: not significant).

F. 4C-seq signal for the distal *Dlk1* CTCF peak on the maternal alleles from ESCs with a deleted CTCF site 2 in the *Meg3* DMR (purple) or their WT counterparts (red) in the entire *Dlk1-Dio3* TAD. The ratio of interactions is provided in-between. The orientation of CTCF sites is indicated below each panel, with an X indicating the deleted CTCF site. The position of the viewpoint and the sub-TADs are indicated above (red box: *Dlk1-Meg3* sub-TAD).

G. Distribution of 4C-seq signal for indicated viewpoints in the *Mirg-Dio3* sub-TAD. In the deletion cells, 3D interactions are reorganized similar to the paternal allele, with increased 4C-seq signal in the *Mirg-Dio3* sub-TAD.

FIGURE S7

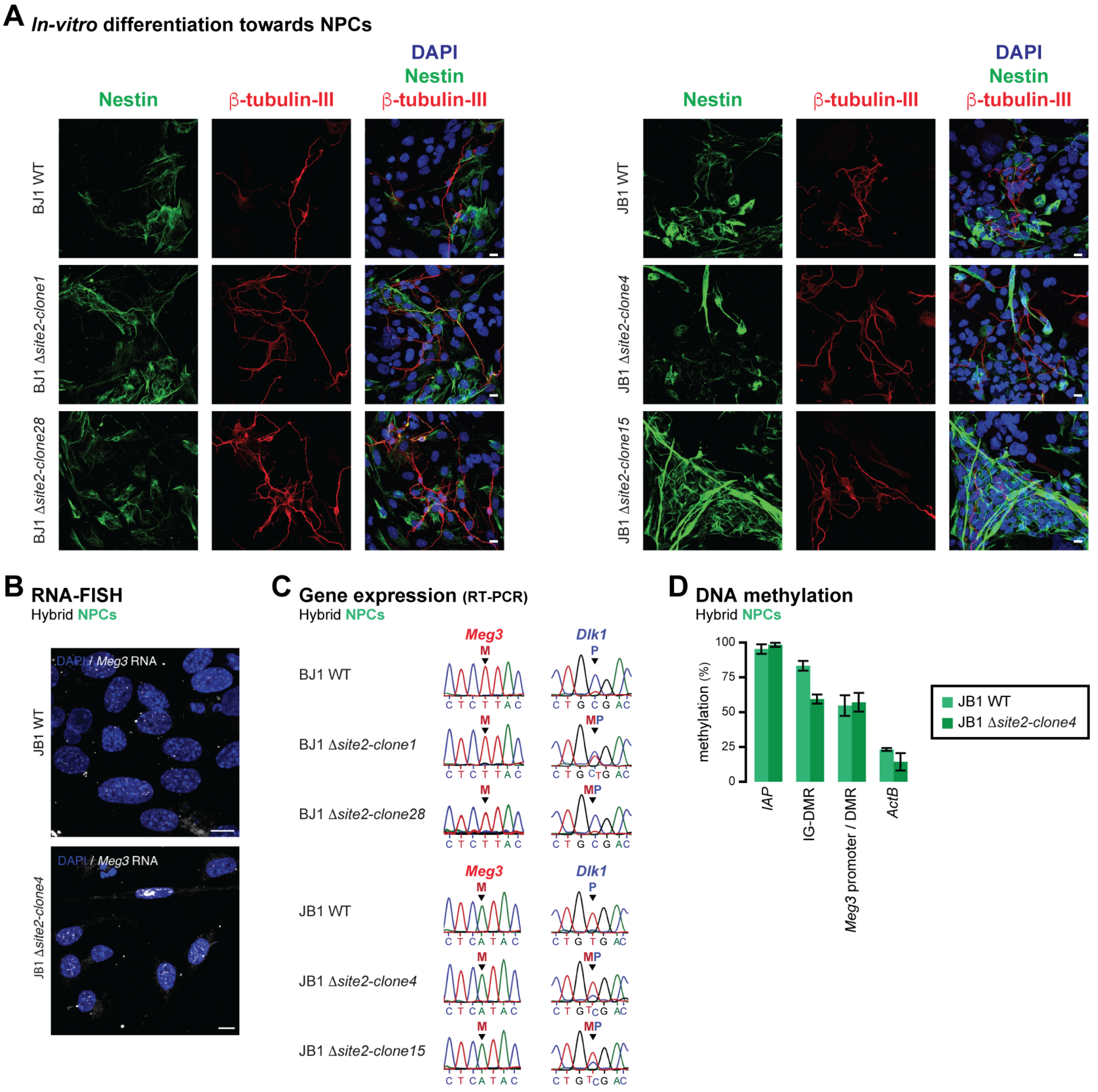
A In-vitro differentiation towards NPCs
Nestin
β-tubulin-III
DAPI
Nestin
β-tubulin-III
BJ1 WT
BJ1 Δsite2-clone1
BJ1 Δsite2-clone28
JB1 WT
JB1 Δsite2-clone4
JB1 Δsite2-clone15
B RNA-FISH
Hybrid NPCs
DAPI / Meg3 RNA
JB1 WT
JB1 Δsite2-clone4
C Gene expression (RT-PCR)
Hybrid NPCs
Meg3
Dlk1
BJ1 WT
BJ1 Δsite2-clone1
BJ1 Δsite2-clone28
JB1 WT
JB1 Δsite2-clone4
JB1 Δsite2-clone15
D DNA methylation
Hybrid NPCs
methylation (%)
100
75
50
25
0
IAP
IG-DMR
Meg3 promoter / DMR
ActB
JB1 WT
JB1 Δsite2-clone4

Figure S7. CTCF binding at site 2 in the *Meg3* DMR is required for correct imprinted activation of *Dlk1*

A. Immunofluorescence staining of Nestin (neuronal progenitors) and Tubulin-β3 (neurons) in NPCs with a deleted CTCF site 2 in the *Meg3* DMR or their WT counterparts. Cells were counterstained with DAPI. Scale bars, 10 μm.

B. Single-molecule inexpensive-FISH (smiFISH) detection of *Meg3* RNA (white) in differentiated JB1-derived NPCs carrying either the WT allele or carrying a deletion in CTCF binding site 2 in the *Meg3* DMR. A single bright RNA accumulation per nucleus, indicating the active transcription site, and additional small spots elsewhere in the nucleoplasm are visible in both WT and mutant cells, as in ESCs (see also Fig. S6C).

C. Maternal allele-specific *Meg3* expression and bi-allelic *Dlk1* activation in *in vitro* differentiated NPCs with a deleted CTCF site 2 in the *Meg3* DMR or their WT counterparts. Determination of allele specificity by Sanger sequencing of RT-PCR products. Parental origin of the SNP that distinguishes the maternal and paternal alleles is indicated.

D. DNA methylation levels as determined by digestion of genomic DNA JB1-derived NPCs (WT or with a deleted CTCF site 2 in the *Meg3* DMR) with *Aci*I at the IG-DMR and the *Meg3* DMR / promoter. Positive control: IAP transposable elements (high levels of methylation) and negative control: *ActB* promoter (low levels of methylation). In agreement with the maintained maternal *Meg3* expression (Fig. S7C), both the IG-DMR and the *Meg3* DMR retain about 50% methylation in the mutant NPCs. The assay was performed as in Figure S1C.

FIGURE S8

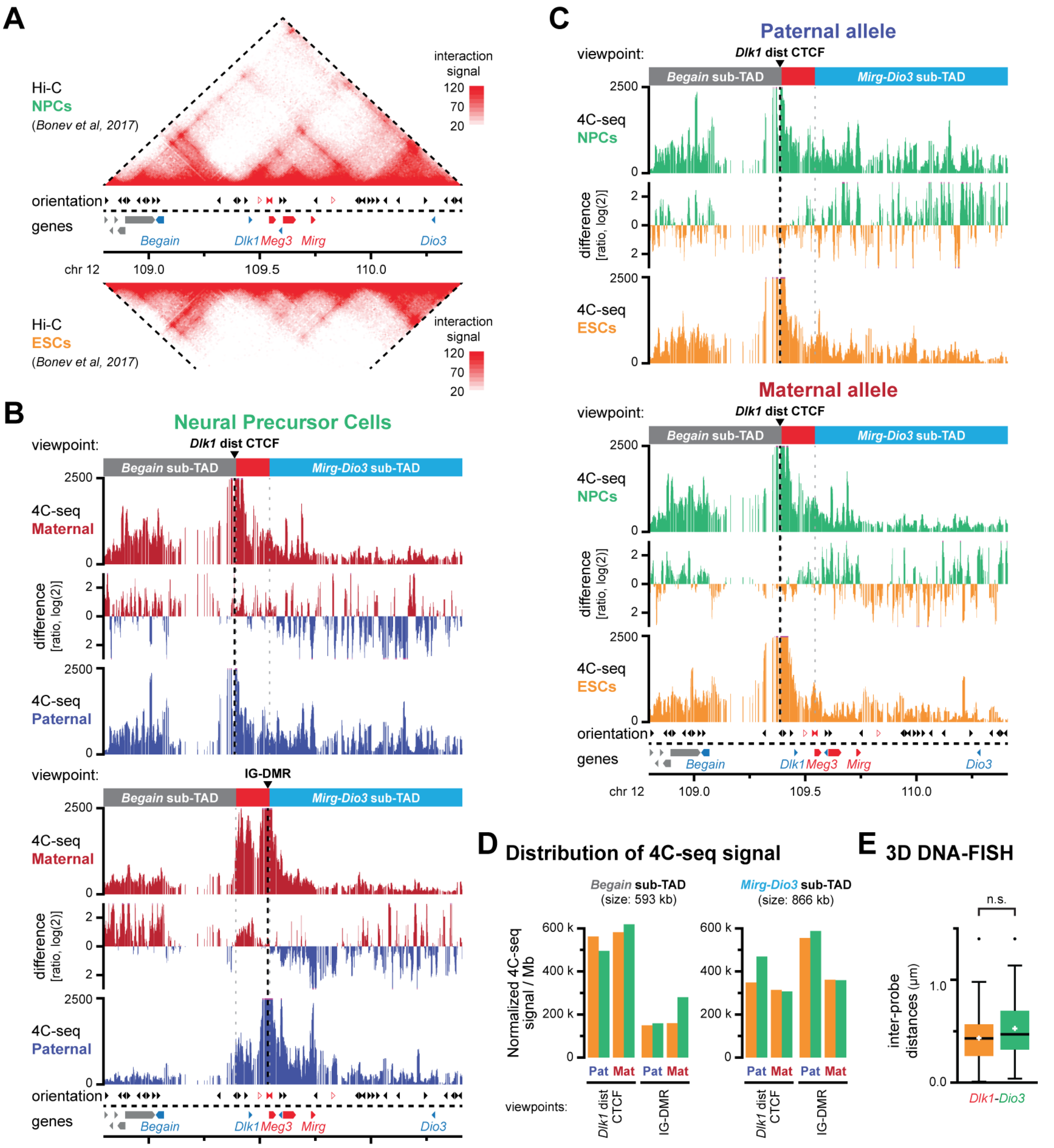
A
Hi-C
NPCs
(Bonev et al, 2017)
interaction signal
120
70
20
orientation
genes
Begain
Dlk1
Meg3
Mirg
Dio3
chr 12
109.0
109.5
110.0
Hi-C
ESCs
B
Neural Precursor Cells
viewpoint:
Dlk1 dist CTCF
Begain sub-TAD
Mirg-Dio3 sub-TAD
4C-seq
Maternal
difference [ratio, log(2)]
Paternal
IG-DMR
C
Paternal allele
Maternal allele
NPCs
ESCs
D
Distribution of 4C-seq signal
Begain sub-TAD (size: 593 kb)
Mirg-Dio3 sub-TAD (size: 866 kb)
Normalized 4C-seq signal / Mb
600 k
400 k
200 k
0
Pat
Mat
viewpoints:
Dlk1 dist CTCF
IG-DMR
E
3D DNA-FISH
n.s.
inter-probe distances (µm)
1.0
0.0
Dlk1-Dio3

<u>Figure S8. Developmental *Dlk1* activation on the maternal chromosome coincides with a mostly stable intra-TAD organisation</u>

A. Reanalysed non-allelic Hi-C signal from NPCs (top) and ESCs (bottom) in the *Dlk1-Dio3* locus. The orientation of CTCF sites identified in ESCs and genes are indicated below with colours indicating allele-specificity. Hi-C data are from reference [1].

B. 4C-seq signal for the distal *Dlk1* CTCF peak (top) and the IG-DMR (bottom) in NPCs cells on the maternal (red) and paternal (blue) allele in the *Dlk1-Dio3* TAD. The ratio of interactions is provided between the patterns. The position of the 4C-seq viewpoints and the sub-TADs are indicated above (red box: *Dlk1-Meg3* sub-TAD).

C. 4C-seq signal for the distal *Dlk1* CTCF peak in *in vitro* differentiated hybrid NPCs (green) and ESCs (orange) on the paternal (top) and maternal (bottom) allele in the *Dlk1-Dio3* TAD. The ratio of interactions is provided between the patterns.

D. Distribution of 4C-seq signal in NPCs and ESCs for indicated viewpoints in the *Begain* and the *Mirg-Dio3* sub-TADs.

E. 3D DNA-FISH distance measurements with fosmid probes (see Fig. 3F) reveal no significant difference in distances between *Dlk1* and *Dio3* in hybrid NPCs and ESCs.

FIGURE S9

Comparison to previous allele-specific studies:

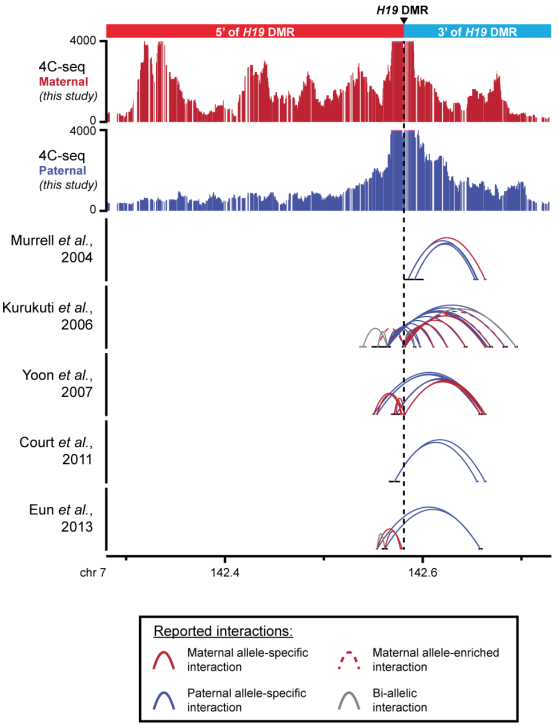

Detection method	Cell type	Restriction fragments	Reference
Endpoint 3C followed by sequencing of SNP *or* co-ChIP with GAL4-tagged *H19* allele.	mouse liver (hybrid)	3C: *KpnI* ChIP: *BamHI*	[3]
Endpoint 3C followed by allelic digestion or sequencing of SNP.	mouse liver (hybrid)	*EcoRI* or *HindIII*	[4]
Endpoint 3C followed by allelic digestion.	mouse fetal muscle and liver (hybrid)	*BamHI and BglII* or *HindIII*	[5]
3C-qPCR comparison between homozygous (bi-allelic) regions and heterozygous deleted regions.	mouse liver (heterozygous deletions)	*BamHI*	[6]
Endpoint 3C followed by allelic digestion.	cultured mouse primary myocytes (hybrid)	*BamHI and BglII*	[7]

Figure S9. Comparison of 3D chromatin organization of the *Igf2-H19* domain to previously published data

Comparison of previously published allele-specific chromatin conformation studies in the context of allele-specific sub-TAD organization at the *Igf2-H19* locus. Allele-specific 4C-seq signal for the *H19* DMR viewpoint and the span of the maternal sub-TADs is indicated above (this study; see Figure 2). Previously identified chromatin interactions and their allelic nature from 5 previous studies, mostly obtained by 3C [3-7], are indicated with color-coded arcs below, with a brief technical description of each study provided on the right. Previous studies were selected only if they allowed the distinction of the parental chromosomes within an otherwise WT setup (i.e. data from cells with a mutated *H19* DMR were not included in this comparison). All previous studies, which probed interactions within a region of up to 150 kb surrounding the *H19* and *Igf2* genes, consistently failed to detect interactions between the sub-domains on either side of the *H19* DMR on the maternal allele, whereas such inter-domain contacts could be detected on the paternal allele.

Table S1. Significance of 4C-seq signal in sub-TADs

Significance of 4C-seq signal for individual sub-TADs or differences between sub-TADs was calculated using an approach previously used to score for differences between regions [8, 9]. The fraction of fragments with increased maternal versus paternal signal was calculated in individual sub-domains versus the remainder of the TAD, or by comparing the score between individual sub-domains. Significance of difference was determined using a G-test of independence.

#: too few instances of signal for reliable calculation of test-statistic.

Color gradient:

$P < 0.001$	$P < 0.01$	$P < 0.05$

A: *Igf2- H19* sub-domains – maternal vs. paternal; ESCs

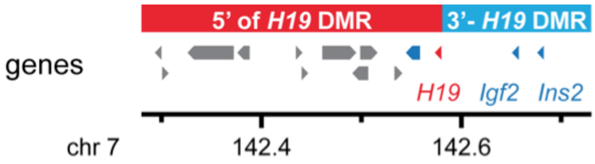

**Viewpoint**	**P-value**<br>**5' of H19 DMR *vs.* 3' of H19 DMR**
Cen *Syt8* CTCF	0.18
Lsp1 CTCF	0.08
H19 DMR – replicate	2.90×10^{-7}
H19 DMR – replicate	4.98×10^{-7}
DS *H19*	0.18
Igf2	0.23

B: *Dlk1-Dio3* domain – maternal vs. paternal; ESCs

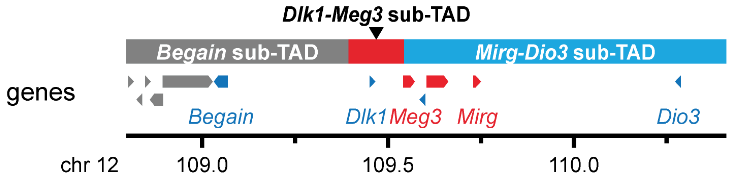

**Viewpoint**	**P-value**		
	***Begain* sub-TAD *vs.* remainder TAD**	***Dlk1-Meg3* sub-TAD *vs.* remainder TAD**	***Mirg-Dio3* sub-TAD *vs.* remainder TAD**
Begain – replicate 1	0.36	0.77	0.69
Begain – replicate 2	2.19×10^{-8}	0.19	0.02
Dlk1 distal CTCF	1.41×10^{-3}	2.07×10^{-3}	0.08
Dlk1 – replicate 1	0.75	2.05×10^{-10}	0.04
Dlk1 – replicate 2	0.52	2.72×10^{-8}	0.07
IG-DMR	6.25×10^{-3}	9.26×10^{-21}	1.11×10^{-4}
Meg3	0.38	3.52×10^{-3}	0.08
Intergenic *Mirg-Dio3*	0.14	0.31	0.92
Dio3 – replicate 1	0.07	#	0.66
Dio3 – replicate 2	0.11	0.13	0.43
Dio3 – replicate 3	0.13	0.64	0.78

C: *Dlk1-Dio3* sub-domains – maternal vs. paternal; ESCs

**Viewpoint**	**P-value**	
	***Dlk1-Meg3* sub-TAD *vs.* *Begain* sub-TAD**	***Dlk1-Meg3* sub-TAD *vs.* *Mirg-Dio3* sub-TAD**
Dlk1 distal CTCF	0.42	4.38×10^{-4}
Dlk1 – replicate 1	1.06×10^{-6}	1.97×10^{-9}
Dlk1 – replicate 2	4.35×10^{-5}	5.54×10^{-8}
IG-DMR	9.67×10^{-9}	9.27×10^{-21}
Meg3	0.57	5.09×10^{-3}

D: *Dlk1-Dio3* domain – maternal vs. paternal; NPCs

**Viewpoint**	**P-value**		
	***Begain* sub-TAD *vs.* remainder TAD**	***Dlk1-Meg3* sub-TAD *vs.* remainder TAD**	***Mirg-Dio3* sub-TAD *vs.* remainder TAD**
Dlk1 distal CTCF	3.48×10^{-6}	3.14×10^{-5}	5.61×10^{-4}
IG-DMR	3.29×10^{-6}	2.84×10^{-24}	6.20×10^{-7}

Table S2. Primers

Genotyping and ChIP-PCR followed by Sanger sequencing

Locus	Sequences (5'-3')
CTCF site in *Meg3* DMR (site 2)	Fwd: CCCCCTCCTACGTCAGTCTA
	Rev: AGGTCACAAGTGTTAGCTGTGTG
CTCF site in *H19* DMR	Fwd: GCGCAAATCACCAGACTTTC
	Rev: CAGGTATCTGACTTATAGGGTTCTG

RT-PCR – hybrid cells (*Mus musculus* C57Bl6 genome)

Gene Name	Sequences (5'-3')	Reference
Dlk1	Fwd: TTGCTCCTGCTGGCTTTC	[10]
	Rev: CCTTGCAGACTCCATTGACA	
Meg3	Fwd: CACAGAAGACGAAGAGCTGGA	[10]
	Rev: GGTAGAGGTGCACAGCAGGT	
Rian	Fwd: CAATGGGTGGATCGTACCTC	[10]
	Rev: GTGCTGCCTCAGTCTTTGTG	
Mirg	Fwd: TCGGCAGTACATACCAGGTG	[10]
	Rev: ACTGATGGCTTCAGGTCAGG	
Gapdh	Fwd: CGTCCCGTAGACAAAATGGT	[10]
	Rev: TGACTGTGCCGTTGAATTTG	
ActB	Fwd: GGCCCAGAGCAAGAGAGGTATCC	[11]
	Rev: ACGCACGATTTCCCTCTCAGC	

ChIP-qPCR – monoparental cells (*Mus musculus* C57Bl6 genome)

Locus	Sequences (5'-3')
Dlk1 prox	Fwd: CTAGGCGGGGCAGGTGTGCT
	Rev: GAAGGCCCCAGAAGGCTCGC
IG *Dlk1-Meg3*	Fwd: AGCACGCTCGCACTGAACCTG
	Rev: GAAGGGAGGAGCAGAGCCCAGAG
Meg3 DMR (site 1)	Fwd: CGCATGATGGCTGCGGCTAGATT
	Rev: AGCCCAGAATGAGGAGGGGGC
Meg3 DMR (site 2)	Fwd: CCCCCTCCTACGTCAGTCTAGCTCT
	Rev: AAGACTCCAATAGCCCAACCACCTGAG
Intergenic *Mirg-Dio3*	Fwd: TCCAGCCTGTGCTTGGCTGC
	Rev: GTGTGAGCTGGGGGCGTGTC

ChIP-qPCR – hybrid cells (*Mus musculus* C57Bl6 genome)

Locus	Sequences (5'-3')	Reference
Meg3 DMR (site 1)	Fwd: CTTTTGCCGTTTCCTTTGTC	[12]
	Rev: AACAAAGGCCACCTCCTCTT	
Meg3 DMR (site 2)	Fwd: CCCCCTCCTACGTCAGTCTA	
	Rev: AGGTCACAAGTGTTAGCTGTGTG	
H19 DMR	Fwd: CATGCTTAGTGGGGTCTGCA	[12]
	Rev: GCCATCAGCGCTATTGTGTG	
Telomeric *H19* (negative control)	Fwd: CGCATGGCACCAGAGAAGTA	[12]
	Rev: TCAGCCCCAAACAGAATCCC	

<u>4C-seq HiSeq2500</u> – monoparental cells (*Mus musculus* C57Bl6 genome)

Viewpoint	Sequences (5’-3’)	
H19 DMR	Fwd:	AATGATACGGCGACCACCGAACACTCTTTCCCTACACGACGCTCTTCCGAT<br>CTATTGTTTGCAGCCCTGAGCC
	Rev:	CAAGCAGAAGACGGCATACGACTCAGACCCCATAAACCAGTGC
Igf2	Fwd:	AATGATACGGCGACCACCGAACACTCTTTCCCTACACGACGCTCTTCCGAT<br>CTCAGCCTCTGTCCTATGCCCC
	Rev:	CAAGCAGAAGACGGCATACGAAACAGCCCCCATACCCCC
Begain	Fwd:	AATGATACGGCGACCACCGAACACTCTTTCCCTACACGACGCTCTTCCGAT<br>CTTCCTGATTACCAAAGACAACAGTCA
	Rev:	CAAGCAGAAGACGGCATACGATTAGCACTGGGGAGAGCTGG
Dlk1	Fwd:	AATGATACGGCGACCACCGAACACTCTTTCCCTACACGACGCTCTTCCGAT<br>CTGGCCTTCCTAACCCTCAGCA
	Rev:	CAAGCAGAAGACGGCATACGAGCTCTCCTGTCCATACGGGT
Meg3	Fwd:	AATGATACGGCGACCACCGAACACTCTTTCCCTACACGACGCTCTTCCGAT<br>CTAACCTATGCTAATGTTTGGATGGGA
	Rev:	CAAGCAGAAGACGGCATACGAGCCAGAAGGACAAACATGTTGC
Intergenic *Mirg-Dio3*	Fwd:	AATGATACGGCGACCACCGAACACTCTTTCCCTACACGACGCTCTTCCGAT<br>CTGGCCTTGACTGGTACTATTTAAAAGC
	Rev:	CAAGCAGAAGACGGCATACGAATCCCCCTCCTCTACAGTGC
Dio3	Fwd:	AATGATACGGCGACCACCGAACACTCTTTCCCTACACGACGCTCTTCCGAT<br>CTCCACTGACTGCTTGGCTCTG
	Rev:	CAAGCAGAAGACGGCATACGACTACAGCTCCAGCTGCTTGC

<u>4C-seq HiSeq2500</u> – hybrid cells (*Mus musculus molossinus* JF1 genome)

Viewpoint	Sequences (5’-3’)	
Cen *Syt8* CTCF	Fwd:	AATGATACGGCGACCACCGAACACTCTTTCCCTACACGACGCTCTTCCGAT<br>CTTGGTCACAGCTCTCCAAGTCT
	Rev:	CAAGCAGAAGACGGCATACGAGGGGCTAGGGTCTACAGCAA
Lsp1 CTCF	Fwd:	AATGATACGGCGACCACCGAACACTCTTTCCCTACACGACGCTCTTCCGAT<br>CTCCCACACCTCATCCAGAGGC
	Rev:	CAAGCAGAAGACGGCATACGATGCCTCACCTGAGTGTGCAT
DS *H19*	Fwd:	AATGATACGGCGACCACCGAACACTCTTTCCCTACACGACGCTCTTCCGAT<br>CTGGGTCCAGAACCCACTTTCTGA
	Rev:	CAAGCAGAAGACGGCATACGATTTCCCCAGAGTAGGGGCTG
Dlk1 distal CTCF	Fwd:	AATGATACGGCGACCACCGAACACTCTTTCCCTACACGACGCTCTTCCGAT<br>CTACTAACCGGGGTCTCTCACG
	Rev:	CAAGCAGAAGACGGCATACGACCTTCAGAACTTTGAGCTAAATAAACCT
IG-DMR	Fwd:	AATGATACGGCGACCACCGAACACTCTTTCCCTACACGACGCTCTTCCGAT<br>CTTCTTCTATCAGCCCTAAGAATCCTGA
	Rev:	CAAGCAGAAGACGGCATACGAATAACCCTGCGGAATGGGTG

<u>4C-seq NextSeq500</u> – hybrid cells (*Mus musculus molossinus* JF1 genome)

Viewpoint	Sequences (5’-3’)	
Dlk1 distal CTCF	Fwd:	AATGATACGGCGACCACCGAGATCTACACTCTTTCCCTACACGACGCTCTTCC<br>GATCTACTAACCGGGGTCTCTCACG
	Rev index 1:	CAAGCAGAAGACGGCATACGAGATCGTGATGTGACTGGAGTTCAGACGTGTG<br>CTCTTCCGATCTCCTTCAGAACTTTGAGCTAAATAAACCT
	Rev index 2:	CAAGCAGAAGACGGCATACGAGATACATCGGTGACTGGAGTTCAGACGTGTG<br>CTCTTCCGATCTCCTTCAGAACTTTGAGCTAAATAAACCT
IG-DMR	Fwd:	AATGATACGGCGACCACCGAGATCTACACTCTTTCCCTACACGACGCTCTTCC<br>GATCTTCTTCTATCAGCCCTAAGAATCCTGA
	Rev index 1:	CAAGCAGAAGACGGCATACGAGATCGTGATGTGACTGGAGTTCAGACGTGTG<br>CTCTTCCGATCTATAACCCTGCGGAATGGGTG
	Rev index 2:	CAAGCAGAAGACGGCATACGAGATACATCGGTGACTGGAGTTCAGACGTGTG<br>CTCTTCCGATCTATAACCCTGCGGAATGGGTG

DNA methylation studies – hybrid cells

Locus	Sequences (5'-3')		Reference
Meg3 DMR (site 2)	Fwd:	CCCCCTCCTACGTCAGTCTA	
	Rev:	AGGTCACAAGTGTTAGCTGTGTG	
ActB	Fwd:	GGCTTTCCGGCTATTGCTA	
	Rev:	CCTCTGGGTGTGGATGTCA	
IAP	Fwd:	CAAATTAAAGAGCTTGCCGAGT	[12]
	Rev:	TAGGGAGAGCGGCTTTTACA	
IG-DMR	Fwd:	CGGTATAGGCCAAGTGGTTTGTAGC	[13]
	Rev:	CTGTTCCGCGAGTCACCCGG	
Meg3 promoter / DMR	Fwd:	GGGTAGGCAGAGCAGCCGGA	[14]
	Rev:	AGGGGTACCCAGCAACCCGG	
Col1a2	Fwd:	AAAGAGAAGGATTGGTCAGAGCAGT	[15]
	Rev:	GCCAAGGGAGGAGACTTAGTTG	
Col9a2	Fwd:	CTCTGGACTTATTTTTATTGGGTATCTTTT	[15]
	Rev:	CAGGGAAGATGGATGTTTAAATACTG	

Table S3. FISH Probes

All coordinates GRCm38/mm10

Fosmid	Locus	Position	Length (bp)
WIBR1-1062J20	mHIDAD CTCF	chr7:142,316,692-142,353,854	37,162
WIBR1-090J20	*H19*	chr7:142,579,083-142,621,420	42,337
WIBR1-399J17	*Igf2*	chr7:142,656,682-142,695,317	38,635
WIBR1-1319N18	Centromeric *Dlk1*	chr12:109,363,854-109,403,919	40,065
WIBR1-1116K16	*Dlk1*	chr12:109,435,989-109,471,592	35,603
WIBR1-1703L18	*Meg3*	chr12:109,539,156-109,578,782	39,626
WIBR1-2409H13	*Dio3*	chr12:110,254,267-110,294,032	39,765

BAC	Locus	Position	Length (bp)
RP23-119N23	Centromeric *Begain*	chr12:108,816,610-109,016,762	200,153
RP23-421C16	*Meg3 / Gtl2*	chr12:109,480,824-109,658,133	177,310
RP23-41I14	Telomeric *Dio3*	chr12:110,431,516-110,659,759	228,244

Supplemental references

1. Bonev B, Mendelson Cohen N, Szabo Q, Fritsch L, Papadopoulos GL, Lubling Y, Xu X, Lv X, Hugnot JP, Tanay A, Cavalli G: **Multiscale 3D Genome Rewiring during Mouse Neural Development.** *Cell* 2017, **171:**557-572 e524.

2. Klein FA, Pakozdi T, Anders S, Ghavi-Helm Y, Furlong EE, Huber W: **FourCSeq: analysis of 4C sequencing data.** *Bioinformatics* 2015, **31:**3085-3091.

3. Murrell A, Heeson S, Reik W: **Interaction between differentially methylated regions partitions the imprinted genes Igf2 and H19 into parent-specific chromatin loops.** *Nat Genet* 2004, **36:**889-893.

4. Kurukuti S, Tiwari VK, Tavoosidana G, Pugacheva E, Murrell A, Zhao Z, Lobanenkov V, Reik W, Ohlsson R: **CTCF binding at the H19 imprinting control region mediates maternally inherited higher-order chromatin conformation to restrict enhancer access to Igf2.** *Proc Natl Acad Sci U S A* 2006, **103:**10684-10689.

5. Yoon YS, Jeong S, Rong Q, Park KY, Chung JH, Pfeifer K: **Analysis of the H19ICR insulator.** *Mol Cell Biol* 2007, **27:**3499-3510.

6. Court F, Baniol M, Hagege H, Petit JS, Lelay-Taha MN, Carbonell F, Weber M, Cathala G, Forne T: **Long-range chromatin interactions at the mouse Igf2/H19 locus reveal a novel paternally expressed long non-coding RNA.** *Nucleic Acids Res* 2011, **39:**5893-5906.

7. Eun B, Sampley ML, Good AL, Gebert CM, Pfeifer K: **Promoter cross-talk via a shared enhancer explains paternally biased expression of Nctc1 at the Igf2/H19/Nctc1 imprinted locus.** *Nucleic Acids Res* 2013, **41:**817-826.

8. Andrey G, Montavon T, Mascrez B, Gonzalez F, Noordermeer D, Leleu M, Trono D, Spitz F, Duboule D: **A switch between topological domains underlies HoxD genes collinearity in mouse limbs.** *Science* 2013, **340:**1195.

9. Noordermeer D, Leleu M, Schorderet P, Joye E, Chabaud F, Duboule D: **Temporal dynamics and developmental memory of 3D chromatin architecture at Hox gene loci.** *Elife* 2014, **3:**e02557.

10. Sanli I, Lalevee S, Cammisa M, Perrin A, Rage F, Lleres D, Riccio A, Bertrand E, Feil R: **Meg3 Non-coding RNA Expression Controls Imprinting by Preventing Transcriptional Upregulation in cis.** *Cell Rep* 2018, **23:**337-348.

11. Leeb M, Pasini D, Novatchkova M, Jaritz M, Helin K, Wutz A: **Polycomb complexes act redundantly to repress genomic repeats and genes.** *Genes Dev* 2010, **24:**265-276.

12. Chatzinikolaou G, Apostolou Z, Aid-Pavlidis T, Ioannidou A, Karakasilioti I, Papadopoulos GL, Aivaliotis M, Tsekrekou M, Strouboulis J, Kosteas T, Garinis GA: **ERCC1-XPF cooperates with CTCF and cohesin to facilitate the developmental silencing of imprinted genes.** *Nat Cell Biol* 2017, **19:**421-432.

13. Kota SK, Lleres D, Bouschet T, Hirasawa R, Marchand A, Begon-Pescia C, Sanli I, Arnaud P, Journot L, Girardot M, Feil R: **ICR noncoding RNA expression controls imprinting and DNA replication at the Dlk1-Dio3 domain.** *Dev Cell* 2014, **31:**19-33.

14. Delaval K, Govin J, Cerqueira F, Rousseaux S, Khochbin S, Feil R: **Differential histone modifications mark mouse imprinting control regions during spermatogenesis.** *EMBO J* 2007, **26:**720-729.

15. Varrault A, Eckardt S, Girard B, Le Digarcher A, Sassetti I, Meusnier C, Ripoll C, Badalyan A, Bertaso F, McLaughlin KJ, et al: **Mouse Parthenogenetic Embryonic Stem Cells with Biparental-Like Expression of Imprinted Genes Generate Cortical-Like Neurons That Integrate into the Injured Adult Cerebral Cortex.** *Stem Cells* 2018, **36:**192-205.
