# Supplementary material for: CTCF modulates allele-specific sub-TAD organization and imprinted gene activity at the mouse Dlk1-Dio3 and Igf2-H19 domains
Source: Genome Biol. 2019 Dec 12;20:272. doi: 10.1186/s13059-019-1896-8 (PMC6909504; doi:10.1186/s13059-019-1896-8)
Supplement: Supplementary file 4 — Additional file 4. Review history. [file 13059_2019_1896_MOESM4_ESM.docx]

**First round**

**Reviewer 1**

This paper investigates the chromatin architecture at two imprinted domains with maternal-specific CTCF binding sites at differentially methylated regions: Igf2-H19, which is actually quite well characterized with respect to architecture, and Dlk1-Meg3, whose architecture is much less well established. The investigators use published datasets, as well as generating their own 4C data and 3D-DNA FISH. In the much larger and more complex Dlk1-Meg3 domain, the investigators identify an intriguing sub-TAD structure. Additionally, they delete one of the two CTCF binding sites in the Meg3 DMR, which results in relaxation of Dlk1 imprinting upon differentiation of the cells into NPCs, but does not disturb the TAD structure. In summary, the in depth allelic analysis of these domains is an important contribution to the field. While it is quite useful that the investigators compared the two imprinted domains with paternal methylation and maternal CTCF binding, it is, however, not surprising that the two domains display different architecture, given that there are many differences between the two.

Specific Points:

1.As stated above, the architecture of the Igf2-H19 has been very well characterized by a number of groups (who are largely unreferenced in this manuscript). It is unclear what new data this manuscript provides with respect to this domain, especially since the work is done in ES cells, which do not express these genes to a significant extent. Additionally, if there are hypotheses to be tested in this work, deletion mutants should have been characterized (as was done for Dlk1-Meg3).

2.What does mHIDAD correspond to?

3.I am a bit confused about the CTCF sites at the Dlk1-Meg3 domain. Does the IG-DMR have CTCF binding sites? The investigators should be more transparent about the differences between the DMRs that are characterized in these two domains. The Igf2-H19 DMR regulates imprinting at the loci, including acquisition of the imprint, whereas the Meg3 DMR is a secondary DMR, with IG-DMR being more central to the locus-specific imprinting.

4.For Dlk1-Meg3 domain, significantly longer distances were measured on the maternal chromosome between all pairs of probes that covered the Dlk1, Dio3 and Begain genes but this was not replicated using 4C-seq in mono-parental ESCs using viewpoints near the Dlk1, Dio3 and Begain promoters. What does the difference in the assays mean?

5.There does not seem to be a difference in sub-TAD structure in the Dlk1-Meg3 domain in the absence of CTCF site 2. However, there is no statistical analysis of the expression in the CTCF site 2 deletion cell lines compared to the wild-type cell lines for the various imprinted genes in the region. Is their expression statistically different? If not, then one cannot conclude that the lack of change in sub-TAD organization in the mutant lines does not correlate with allelic expression.

6. In the discussion, the authors state "In contrast, at the Dlk1-Dio3 domain, the maternal Dlk1-Meg3 sub-TAD further clusters the repressed Dlk1 gene and its identified regulatory elements (Fig. 3G). The necessity for this maternal-specific clustering, mediated by CTCF binding to the Meg3 DMR, was shown by deletion of CTCF binding site 2, which resulted in a more relaxed imprinting of the Dlk1 gene, with developmental activation now occurring on both parental chromosomes." The data show that CTCF binding site 2 is required for complete repression of the maternal Dlk1 gene but there is no evidence presented here that the sub-TAD structure is required for this. The experiments point to associations and not causality.

7. Is it possible that CTCF binding site 1 in the Meg3 DMR is maintaining the sub-TAD structure in the advance of CTCF binding site 2? I am unclear how close these binding sites are.

**Reviewer 2**

This manuscript by Lleres et al. describes an important study to understand the chromosomal topology driving regulation at the imprinted H19/Igf2 and Dlk1-Dio3 loci. The study combines high resolution 4C and DNA-FISH analyses to look at structures at both loci. These follow up on significant numbers of 3C studies already published describing the H19/Igf2 locus. In addition, this study describes new genetic analyses that directly address the importance of CTCF binding sites at the Dlk1-Dio3 locus.

H19/Igf2 and Dlk1-Dio3 loci are both relatively rare imprinted regions in that regulation is dependent upon a paternally methylated ICR. This new study demonstrates other mechanisms in common. CTCF binding at the methylated ICR does not regulate structures at the TAD level but rather organizes sub-TADs especially by restricting interactions on the maternal chromosome.

The study is very well done. The writing and Figures are particularly clear. The Discussion is interesting and brings together diverse findings in the field. The paper will be of interest and importance to the field genomic imprinting and also to gene regulation. The study takes advantage of the unusual features of imprintin and the already extensive characterization of the these two loci and devises an innovative and carefully executed experimental plan that uncovers features of gene regulation that are very likely to of wide application and significance.

I have only specific comments/suggestions that might improve the paper and make it more accessible to other readers.

1. The Discussion includes an interesting and cogent examination of the merits of including both FISH and 4C analyses and even points out that these results may appear contradictory or paradoxical. I think it would be useful if the authors had briefly raised this issue in the Results (page 4 presentation of Figure 4). Instead the authors present these the 4C and FISH as if they are entirely coherent together. It would be a much easier and more productive read of the paper to know that a full discussion was coming.

2. The Discussion does a great job of addressing many key previous results. I think a couple of more citations would be useful to the reader. First, I don't think there is any evidence that H19 lncRNA plays a role in establishing expression patterns (Jones et al. 1998). This fact might be usefully inserted during the discussion of Meg3. Second, a paper by Eun et al (2013) includes 3C studies from the perspective of the shared enhancer element that strongly support the models in this paper of CTCF binding on the maternal ICR superimposing sub-TAD structures on the locus.

**Reviewer 3**

This study investigates the relationship between imprinting and genome organization as profiled by allele-specific methylation, expression and conformation capture (4C) experiments. One of the two loci studied is the classical example of imprinting with a large number of publications on it (only a small subset of which are referenced). For the other loci, this work provides mechanistic studies demonstrating the importance of allele-specific CTCF binding for establishing imprinted gene expression.

Overall, the manuscript is valuable to the field and presents interesting observations. However, these observations are not always quantified properly, especially in regards to 4C data, which raises questions about the robustness of the reported results. Proper computational tools and statistical tests have to be employed before the work is acceptable for publication. The addition of FISH experiments strengthens the conclusions from 4C/Hi-C data (the differences are not striking but it is expected given the difficulties with FISH experiments for probes that are not too far away). Another technical comment is that since TAD and sub-TAD level structures are of interest, the choice of 4C-seq as the conformation capture method does not seem to be ideal. Region capture Hi-C could have been a better choice but given its expense and other concerns I would not make it a requirement for publication even though its addition would have significantly strengthened the paper.

Major

1. I find it a bit difficult to interpret what the conclusion is for cases such as Fig 3 when FISH results do not agree with 4C-seq. This is not an easy task and there are specific papers explaining why and how these differences can be reconciled but there is no discussion of that in this manuscript. Some sections, including the discussion of Fig 3(A-E), end up being left as inconclusive, which makes it hard to understand what really is the biological significance.

2. I suggest that the authors include one or two concluding sentences after each results subsection. This is done for some subsections but not all. Given the length of the manuscript and the number of different results presented, this would make the manuscript much easier to follow.

3. It is not clear to me how the Figure 5 and NPCs fit into this story or whether it is central. This could be move to the supplement in my opinion.

4. For the 4C data analysis, appropriate computational tools should be used to identify interacting regions and significant differences between compared conditions. FourCSeq, 4Cker are examples of such tools though admittedly no single tool is a perfect solution to 4C analysis, specifically for the differential analysis.

Minor

- Heatmap color scales or ranges should be adjusted appropriately. For instance, for Fig 1A, it is really difficult to see the TAD organization as everything looks dark red. If figure is extended by flanking regions this could be fixed. Also, color bars will have to be provided with appropriate legends for each heatmap.

- Page 2, right column, lines 10-15: Please mention that the Hi-C data used here is from a reference study (Bonev et al 2017).

- Page 2, right column, lines 38-42: It is not clear to me exactly which visual this sentence is supposed to refer to. There is nothing in figure 1 to this effect.

-	Chromosome segment of Igf2-H19 TAD (Figs. 1(A) and S2(A), indicated as chr7:142.4-142.6 Mb) do not match with the segment provided in S2(C) (chr7:141-142 Mb). According to this label, neighboring (+1) TAD should be actual Igf2-H19 TAD segment.

-	Page 4, left column, lines 26-32: The span of each of "the 4 bi-allelic clusters of CTCF" in Fig 1A has to be highlighted clearly.

-	Page 4, left column, lines 49-50: "non-allele specific TAD" is a confusing phrase since Hi-C data (Bonev 2017) used here does not tell us whether these TADs are allele-specific or not. I suggest rephrasing this section to say "... domains identified by Hi-C do not exhibit allelic differences as measured by allele-specific 4C".

-	Fig. 2(A), S3(A-B): Better visualization of differences potentially using arcs may help highlight allelic differences detected from 4C.

-	Page 8, left column, line 40: Fig. S1A does not seem to be the correct reference

**Response to reviewers**

***Reviewer #1:***

This paper investigates the chromatin architecture at two imprinted domains with maternalspecific

CTCF binding sites at differentially methylated regions: *Igf2-H19*, which is actually

quite well characterized with respect to architecture, and *Dlk1-Meg3*, whose architecture is

much less well established. The investigators use published datasets, as well as generating

their own 4C data and 3D-DNA FISH. In the much larger and more complex *Dlk1-Meg3*

domain, the investigators identify an intriguing sub-TAD structure. Additionally, they delete

one of the two CTCF binding sites in the Meg3 DMR, which results in relaxation of Dlk1

imprinting upon differentiation of the cells into NPCs, but does not disturb the TAD structure.

In summary, the in depth allelic analysis of these domains is an important contribution to the

field. While it is quite useful that the investigators compared the two imprinted domains with

paternal methylation and maternal CTCF binding, it is, however, not surprising that the two

domains display different architecture, given that there are many differences between the

two.

*We thank the reviewer for agreeing that our study shows important insights to the field,*

*and for providing useful and constructive comments. Below, we have provided detailed*

*responses to each comment.*

1. As stated above, the architecture of the *Igf2-H19* has been very well characterized by a

number of groups (who are largely unreferenced in this manuscript). It is unclear what

new data this manuscript provides with respect to this domain, especially since the work is

done in ES cells, which do not express these genes to a significant extent. Additionally, if

there are hypotheses to be tested in this work, deletion mutants should have been

characterized (as was done for *Dlk1-Meg3*).

*Earlier studies used 3C technology, or similar ‘one-vs-few’ assays, to identify interactions*

*within a 150 kb region around the Igf2 and H19 genes. Based on these studies, models*

*of 3D organization were proposed whereby different regulatory elements (promoters,*

*enhancers, DMRs) engage in various combinations of allele-specific chromatin loops.*

*The interactions that were probed in these studies tended to have little overlap, which*

*initially made us hesitate to discuss these results within a comprehensive context.*

*As correctly suggested by the reviewer, we should compare our data to these previous*

*studies. For this reason, we have now carefully mapped back the results from the 5 most*

*relevant 3C studies (i.e. where interactions could be scored in an allele-specific manner*

*in WT cells). We have added these results in the newly added Figure S9 (Additional File*

*1) and we dedicate a paragraph in the Discussion to the comparison of our 4C-seq*

*results with these previous studies (from line 349). Moreover, in the introduction we now*

*mention these same 5 studies as well (indicating a detailed discussion will follow later*

*on).*

*As the reviewer will hopefully agree, this comparison provides both support for our*

*model and nuance to the interpretation of previous 3C results:*

*(1) The ‘one-vs-all’ nature of 4C-seq allows us to identify the loops between the various*

*(allele-specific) CTCF sites that appear to be the key determinants for the sub-TAD*

*organization at the domains. Until now, these loops were not reported in detail, as they*

*are located outside of the domain previously probed by 3C.*

*(2) The previous 3C data should not be interpreted in the context of allele-specific loops,*

*but rather as an allele-specific presence or absence of sub-domains*

*(3) The H19 DMR acts as the allele-specific border, determining if contacts are blocked*

*between the two sub-domains (maternal allele only).*

*Also, the Reviewer asks about the relevance of studying ES cells (ESCs), given that Igf2*

*and H19 are inactive in these cells. The same is true for the Dlk1-Dio3 domain, where*

*Dlk1 is barely expressed in ESCs, but becomes transcriptionally activated upon ESC*

*differentiation only (Sanli et al. 2018). Importantly, this situation provides us the unique*

*opportunity to determine structural features that precede the allele-specific activation of*

*imprinted genes and might therefore contribute to this process, rather than being a*

*consequence of differential gene expression.*

*As part of our 4C studies on hybrid ES cells differentiated into NPCs, we generated data*

*at the Igf2-H19 locus, and we prepared a figure for the Reviewer (see attached Reviewer*

*figure 1). At the Igf2-H19 domain, chromatin architecture between ESCs (orange) and*

*NPCs (green) remains highly similar, with the pronounced allelic differences persisting*

*upon neuronal differentiation. Based on the study by Bouschet et al. 2017 (referenced in*

*the manuscript) a considerable activation of both genes can be expected in NPCs. Yet,*

*Igf2 expression does not appear to show a strong allelic bias in NPCs, which is expected*

*given that in many neural cells and tissues, this gene is not imprinted. For this reason,*

*we chose not to include these data. If, however, the Reviewer wishes us to include these*

*data, we will do so.*

2. What does mHIDAD correspond to?

*HIDAD stands for “H19/Igf2 Distal Anchor Domain”, which was identified and named*

*previously in human cells (Rao et al., Cell. 2014; 159:1665). We now clarify that*

*mHIDAD is the mouse homologue of this element, and write out the name in full, in line*

*159.*

3. I am a bit confused about the CTCF sites at the *Dlk1-Meg3* domain. Does the IG-DMR

have CTCF binding sites? The investigators should be more transparent about the

differences between the DMRs that are characterized in these two domains. The *Igf2-H19*

DMR regulates imprinting at the loci, including acquisition of the imprint, whereas the

Meg3 DMR is a secondary DMR, with IG-DMR being more central to the locus-specific

imprinting.

*We thank the reviewer for pointing out that the manuscript benefits from a better*

*description of the complex imprinting mechanism at the locus. In the Introduction and the*

*Results sections we now describe these DMRs better. Moreover, we have modified*

*Figure 1C such that the binding of CTCF to the IG-DMR (not bound) and Meg3 DMR*

*(maternal specific bound) can be directly assessed.*

4. For Dlk1-Meg3 domain, significantly longer distances were measured on the maternal

chromosome between all pairs of probes that covered the Dlk1, Dio3 and Begain genes

but this was not replicated using 4C-seq in mono-parental ESCs using viewpoints near the

Dlk1, Dio3 and Begain promoters. What does the difference in the assays mean?

*We thank the reviewer (and the other reviewers) for pointing out that our discussion of*

*this discrepancy between FISH and 4C-seq data in the Discussion section was not*

*sufficiently clear. From line 390, we now discuss in more detail previous efforts to better*

*understand the different aspects of chromatin organization that are characterized by*

*FISH and 4C-seq. This is followed by a discussion how the apparently paradoxical*

*observations may actually help to define a more refined chromatin architecture, that not*

*only provides insights into the presence of sub-domains, but also how those domains*

*may be positioned among each other.*

5. There does not seem to be a difference in sub-TAD structure in the Dlk1-Meg3 domain in

the absence of CTCF site 2. However, there is no statistical analysis of the expression in

the CTCF site 2 deletion cell lines compared to the wild-type cell lines for the various

imprinted genes in the region. Is their expression statistically different? If not, then one

cannot conclude that the lack of change in sub-TAD organization in the mutant lines does

not correlate with allelic expression.

*Based on the reviewer’s comment, we realize that our description of the effect on the*

*Dlk1-Meg3 sub-TAD structure upon CTCF site 2 may not have been sufficiently clear.*

*The deletion has a considerable effect, with the maternal allele adopting a much more*

*paternal-like configuration (i.e. a strong reduction in the separation between the Dlk1-*

*Meg3 and Mirg-Dio3 sub-TADs. To make this result more easily accessible, we have*

*added a cartoon to summarize these findings to Figure 4E, combined with a more to the*

*point summary of the result in the text.*

*The reviewer correctly pointed out that a statistical analysis of the gene expression data*

*strengthens the credibility of the data. We have now assessed the differences in gene*

*expression upon deletion of CTCF site 2 using a 2-tailed t-test. This analysis confirms*

*that there is no significant change in Meg3 (Figure 4A), Rian or Mirg (Figure S6E)*

*expression. This analysis shows that the expression of this polycistronic transcription*

*unit consisting of the multiple lncRNAs is not altered. To further emphasize this*

*important point, we have added a new panel to Figure S6C to show that the Meg3*

*lncRNA has an unaltered nuclear localization as well, as reflected by its predominant*

*signal at its transcription site.*

*As part of our improved statistical analysis, we revisited all panels containing error bars.*

*We have now modified all error bars such that they consistently depict the SEM.*

*Moreover, for all RT-qPCR data statements on statistical differences have been added*

*(Figure 4F, G).*

6. In the discussion, the authors state "In contrast, at the Dlk1-Dio3 domain, the maternal

Dlk1-Meg3 sub-TAD further clusters the repressed Dlk1 gene and its identified regulatory

elements (Figure 3G). The necessity for this maternal-specific clustering, mediated by

CTCF binding to the Meg3 DMR, was shown by deletion of CTCF binding site 2, which

resulted in a more relaxed imprinting of the Dlk1 gene, with developmental activation now

occurring on both parental chromosomes." The data show that CTCF binding site 2 is

required for complete repression of the maternal Dlk1 gene but there is no evidence

presented here that the sub-TAD structure is required for this. The experiments point to

associations and not causality.

*We agree with reviewer that we should be cautious to conclude that the sub-TAD,*

*though altered by the CTCF site-2 deletion, is not necessarily required for the complete*

*repression of the Dlk1 gene on the maternal chromosome. We have rewritten the*

*Discussion to bring it in line with its description in the Results section: “In contrast, at the*

*Dlk1-Dio3 domain, the CTCF-structured maternal Dlk1-Meg3 sub-TAD further clusters*

*the repressed Dlk1 gene and its identified regulatory elements (Figure 3G). The*

*necessity of CTCF binding to the Meg3 DMR, associated with the maternal-specific*

*clustering of the sub-TAD, was shown by deletion of CTCF binding site 2, which resulted*

*in a more relaxed imprinting of the Dlk1 gene, with developmental activation now*

*occurring on both parental chromosomes”.*

7. Is it possible that CTCF binding site 1 in the Meg3 DMR is maintaining the sub-TAD

structure in the advance of CTCF binding site 2? I am unclear how close these binding

sites are.

*As previously mentioned, the structure of the Dlk1-Meg3 sub-TAD structure is*

*considerably reorganized upon deletion of CTCF site 2 alone. Such a change may be*

*expected due to the orientation of this strongly bound CTCF site, as it is directed towards*

*Dlk1.*

*We consider it less likely that site 1 will have a major effect on Dlk1-Meg3 sub-TAD*

*structure, as it both relatively weakly bound and directed towards the distal part of the*

*domains. As part of this project, we have tried to obtain cells with CTCF site 1 deletions*

*as well (which is located in the Meg3 promoter). However, in all three ES cell clones*

*where this site was deleted on the maternal chromosome, the Meg3 transcript could not*

*be detected anymore (data not shown). Considering the known importance of correct*

*Meg3 expression in our system, particularly for Dlk1 imprinting (Sanli et al. 2018), we*

*considered these cells unsuited to draw conclusions in the context of the current*

*structural study. We are continuing our efforts using more complex approaches for future*

*studies, but we think the discussion of site 2 is sufficient to make our point about sub-*

*TAD organization of the Dlk1-Dio3 locus.*

*Regarding the distance between site 1 and 2; the two sites are about 900 bp apart. To*

*better emphasize this short distance, we have modified Figure 1C and we have repeated*

*this distance in the section about the CTCF-site deletion.*

***Reviewer #2:***

This manuscript by Lleres et al. describes an important study to understand the

chromosomal topology driving regulation at the imprinted H19/Igf2 and Dlk1-Dio3 loci. The

study combines high resolution 4C and DNA-FISH analyses to look at structures at both

loci. These follow up on significant numbers of 3C studies already published describing the

H19/Igf2 locus. In addition, this study describes new genetic analyses that directly address

the importance of CTCF binding sites at the Dlk1-Dio3 locus.

H19/Igf2 and Dlk1-Dio3 loci are both relatively rare imprinted regions in that regulation is

dependent upon a paternally methylated ICR. This new study demonstrates other

mechanisms in common. CTCF binding at the methylated ICR does not regulate structures

at the TAD level but rather organizes sub-TADs especially by restricting interactions on the

maternal chromosome.

The study is very well done. The writing and Figures are particularly clear. The Discussion

is interesting and brings together diverse findings in the field. The paper will be of interest

and importance to the field genomic imprinting and also to gene regulation. The study takes

advantage of the unusual features of imprinting and the already extensive characterization of

the these two loci and devises an innovative and carefully executed experimental plan that

uncovers features of gene regulation that are very likely to of wide application and

significance.

I have only specific comments/suggestions that might improve the paper and make it more

accessible to other readers.

*We thank the reviewer for his/her enthusiasm for our study and manuscript, and for*

*providing useful suggestions. Below, we have provided detailed responses to each*

*remark.*

1. The Discussion includes an interesting and cogent examination of the merits of including

both FISH and 4C analyses and even points out that these results may appear

contradictory or paradoxical. I think it would be useful if the authors had briefly raised this

issue in the Results (page 4 presentation of Figure 4). Instead the authors present these

the 4C and FISH as if they are entirely coherent together. It would be a much easier and

more productive read of the paper to know that a full discussion was coming.

*We agree with this reviewer (and reviewer 1 and 3) that the apparent contradictory and*

*paradoxical differences between 4C-seq and FISH should be acknowledged better in the*

*results section. In line 170, we now mention that the apparent incompatibility will be*

*discussed in the Discussion section. Moreover, to further reinforce this aspect of the*

*manuscript, we have now expanded this section in the Discussion section (from line*

*390).*

2. The Discussion does a great job of addressing many key previous results. I think a

couple of more citations would be useful to the reader. First, I don't think there is any

evidence that H19 lncRNA plays a role in establishing expression patterns (Jones et al.

1998). This fact might be usefully inserted during the discussion of Meg3. Second, a

paper by Eun et al (2013) includes 3C studies from the perspective of the shared

enhancer element that strongly support the models in this paper of CTCF binding on the

maternal ICR superimposing sub-TAD structures on the locus.

*We thank the reviewer for reminding us of these studies that further support our findings.*

*The study by Jones et al. (Genes Dev 1998), showing that the H19 RNA is indispensable*

*for imprinted activity of the Igf2 gene) is now discussed in the Discussion section (line*

*387). Upon suggestion by Reviewer 1, we have added a systematic comparison of our*

*findings to previous 3C data (newly added Figure S9 in Additional File 1). In this figure,*

*the previous findings by Eun et al. (NAR 2013) are shown to fit very well within the*

*context of our model for sub-TAD organization.*

*Reviewer #3:*

This study investigates the relationship between imprinting and genome organization as

profiled by allele-specific methylation, expression and conformation capture (4C)

experiments. One of the two loci studied is the classical example of imprinting with a large

number of publications on it (only a small subset of which are referenced). For the other loci,

this work provides mechanistic studies demonstrating the importance of allele-specific CTCF

binding for establishing imprinted gene expression.

Overall, the manuscript is valuable to the field and presents interesting observations.

However, these observations are not always quantified properly, especially in regards to 4C

data, which raises questions about the robustness of the reported results. Proper

computational tools and statistical tests have to be employed before the work is acceptable

for publication. The addition of FISH experiments strengthens the conclusions from 4C/Hi-C

data (the differences are not striking but it is expected given the difficulties with FISH

experiments for probes that are not too far away). Another technical comment is that since

TAD and sub-TAD level structures are of interest, the choice of 4C-seq as the conformation

capture method does not seem to be ideal. Region capture Hi-C could have been a better

choice but given its expense and other concerns I would not make it a requirement for

publication even though its addition would have significantly strengthened the paper.

*We thank the reviewer for agreeing that our study provides interesting insights, and for*

*giving useful and constructive suggestions. Below, we have provided detailed responses*

*to each suggestion.*

*Regarding the choice for 4C-seq over region capture Hi-C, we agree with the reviewer*

*that such data would have been an exciting addition. An allele-specific analysis at the*

*same resolution as our 4C-seq in hybrid cell lines would be complicated though, if not*

*impossible, as SNPs are relatively infrequent and unevenly spread. As in-vitro*

*differentiation can only be done in hybrid cells, we would be limited in our analysis to*

*embryonic stem cells (i.e. the bi-allelic states of imprinted loci in mono-parental cells*

*precludes such experiments).*

1. I find it a bit difficult to interpret what the conclusion is for cases such as Fig 3 when FISH

results do not agree with 4C-seq. This is not an easy task and there are specific papers

explaining why and how these differences can be reconciled but there is no discussion of

that in this manuscript. Some sections, including the discussion of Fig 3(A-E), end up

being left as inconclusive, which makes it hard to understand what really is the biological

significance.

*We thank the reviewer (and the other reviewers) for mentioning the need to comment in*

*more detail on the differences between our 4C-seq and DNA-FISH data. From line 390,*

*we now discuss in more detail previous efforts to better understand the different aspects*

*of chromatin organization that are characterized by FISH and 4C-seq. This is then*

*followed by a discussion how the apparently paradoxical observations may actually help*

*to define a more refined chromatin architecture, that not only provides insights into the*

*presence of sub-domains, but also how those domains may be positioned among each*

*other.*

2. I suggest that the authors include one or two concluding sentences after each results

subsection. This is done for some subsections but not all. Given the length of the

manuscript and the number of different results presented, this would make the manuscript

much easier to follow.

*We thank the reviewer for this suggestion. We now consistently provide a small*

*summary after each paragraph in the results section.*

3. It is not clear to me how the Figure 5 and NPCs fit into this story or whether it is central.

This could be move to the supplement in my opinion.

*The main emphasis of this study is on mESCs, in which the imprinted genes at the Igf2-*

*H19 domain are barely expressed (Additional file 3). Similarly, at the Dlk1-Dio3 domain*

*in mESCs, the Dlk1 gene remains inactive. Yet, this gene (whose correct imprinted*

*activation is of great importance for development) will be allele-specifically activated*

*upon ESC differentiation towards NPCs (see e.g. Bouschet et al. 2017 and Sanli et al.*

*2018, both referenced in the manuscript). As such, this system provided us with the*

*unique opportunity to determine if the structural features that precede the allele-specific*

*activation of this imprinted gene stably contribute to allelic activation, or if this includes a*

*further (allele-specific) restructuration of chromatin architecture.*

*Although the lack of 3D reorganization may be considered a negative result, it fits with*

*the idea of (sub-)TADs acting as relatively stable regulatory neighborhoods. These*

*unique allele-specific sub-TADs therefore function as expected on the maternal allele.*

*We think that this finding is important for the understanding of the sub-TAD function in*

*imprinted gene activation, and thus would like to keep this figure, and all findings related*

*to NPCs, included as part of the main manuscript.*

4. For the 4C data analysis, appropriate computational tools should be used to identify

interacting regions and significant differences between compared conditions. FourCSeq,

4Cker are examples of such tools though admittedly no single tool is a perfect solution to

4C analysis, specifically for the differential analysis.

*The reviewer raises an interesting issue here. First, we realize we may have not*

*sufficiently acknowledged that we used the same statistical approach to score for*

*differences in several other highly cited publications (i.e. Andrey et al, Science 2013 and*

*Noordermeer et al, eLife 2014). We have now mentioned these publications in the*

*Methods section and in Table S1 (Additional data file 1). Although we agree that no*

*approach is perfect, we believe that our approach is relatively unbiased, as it looks for*

*significant differences between regions within the same data set (with the borders*

*between all regions coinciding with CTCF peaks). We consider such an approach*

*relatively robust against technical variation, particularly at the data-dense regions close*

*to the viewpoint.*

*Next, to further reinforce our observations, we applied FourCSeq, 4Cker and the recently*

*published PeakC (Geeven et al. 2018 NAR) to our data. As discussed below, this*

*provides mixed results:*

*Using FourCSeq on our H19 DMR viewpoint (Igf2-H19 domain), all previously mentioned*

*maternal-specific upstream CTCF peaks are identified as significantly different between*

*the maternal and paternal chromosomes. We have added this result as a separate track*

*to Figure 2A and S3A, and adjusted our discussion of the results accordingly (see also*

*point 11).*

*For the Dlk1-Dio3 domain, none of the tools provided satisfactory results. A summary of*

*our analysis is provided in the attached Reviewer figure 2. As may be appreciated from*

*this figure, FourCSeq generates very sparse data at this locus. This could be expected,*

*as the chromatin organization of this locus, probed using our 4C-seq viewpoints,*

*appears not to include many DNA loops. It is interesting to note though, that for the*

*maternal IG-DMR viewpoint the interaction with the CTCF site upstream of the Dlk1*

*gene is called as significant.*

*4Cker, using the near-bait setting, generates data that covers a very large fraction of the*

*domain, both on the maternal and paternal chromosome. For individual viewpoints, it is*

*difficult to extract meaningful data from this analysis. Interestingly, by combining the*

*maternal or paternal signal for the four viewpoints together, we do generate a pattern*

*that closely resembles our 4C-seq data (bottom section; “Combined 4Cker”). Further*

*taking the ratio between these two tracks (“4Cker Cum Sum track”), albeit a rather*

*artificial analysis, thus provides further statistical support for our identified domains and*

*our previous statistical analysis.*

*In summary: we have added the results of the FourCSeq analysis of the Igf2-H19*

*section the manuscript. For the Dlk1-Dio3 locus, the results are either very scarce*

*(FourCSeq, PeakC) or require the pooling of all four viewpoints to obtain a meaningful*

*result. For these reasons we prefer not to include these data, but rather to rely on our*

*initial analysis (Table S1).*

5. Heatmap color scales or ranges should be adjusted appropriately. For instance, for Fig

1A, it is really difficult to see the TAD organization as everything looks dark red. If figure is

extended by flanking regions this could be fixed. Also, color bars will have to be provided

with appropriate legends for each heatmap.

*The reviewer is correct that certain representations can be improved. We have increased*

*the range for the local Igf2-H19 matrix, such that more detail is visible (Figs. 1A, 2A,*

*S2A). For all Hi-C matrices, a gradient legend has been added (Figs. 1A-B, 2A, S2A-D,*

*S5E, S8A).*

6. Page 2, right column, lines 10-15: Please mention that the Hi-C data used here is from a

reference study (Bonev et al 2017).

*We have moved the reference to the location as suggested by the reviewer.*

7. Page 2, right column, lines 38-42: It is not clear to me exactly which visual this sentence is

supposed to refer to. There is nothing in figure 1 to this effect.

*This sentence refers to panel 1B, where three (putative) allele-specific CTCF binding*

*sites are highlighted by arrows in the materal ChIP-seq track. To avoid confusion, we*

*now provide more guidance to the reader in line 100: “(Figure 1b, arrows in maternal*

*ChIP-seq track…)”.*

8. Chromosome segment of Igf2-H19 TAD (Figs. 1(A) and S2(A), indicated as chr7:142.4-

142.6 Mb) do not match with the segment provided in S2(C) (chr7:141-142 Mb).

According to this label, neighboring (+1) TAD should be actual Igf2-H19 TAD segment.

*We thank the reviewer for spotting this inconsistency. The coordinates in Figure S2C*

*have been corrected*.

9. Page 4, left column, lines 26-32: The span of each of "the 4 bi-allelic clusters of CTCF" in

Fig 1A has to be highlighted clearly.

*We have modified Figs. 1A-B to better indicate the span of the CTCF clusters.*

10. Page 4, left column, lines 49-50: "non-allele specific TAD" is a confusing phrase since

Hi-C data (Bonev 2017) used here does not tell us whether these TADs are allelespecific

or not. I suggest rephrasing this section to say "... domains identified by Hi-C do

not exhibit allelic differences as measured by allele-specific 4C".

*We agree with the reviewer that this title may be confusing. We have replaced it with a*

*slightly shortened version of the reviewer’s suggestion: “The Igf2-H19 and Dlk1-Dio3*

*domains are located within TADs that do not exhibit allelic differences, as measured by*

*allele-specific 4C-seq”.*

11. Fig. 2(A), S3(A-B): Better visualization of differences potentially using arcs may help

highlight allelic differences detected from 4C.

*We agree with the reviewer that the significance of differences can be visualized better.*

*Based on this reviewer’s suggestion (point 4), we have used FourCSeq to identify*

*significant differences. We added a dedicated track below the ratio track to highlight*

*this result. To not further complicate this figure, we prefer this over adding Arcs as a*

*further level of information.*

12. Page 8, left column, line 40: Fig. S1A does not seem to be the correct reference

*We thank the reviewer for spotting this incorrect reference. We have corrected it to*

*Figure S2A.*

**2^nd^ Round**

**Reviewer 1**

The authors have presented a clarified version of the paper and addressed many of the concerns of the reviewers. Nevertheless, there are still aspects of this paper that require confirmation. 

Line 95-97: “For the Igf2- H19 domain, we detected maternal allele-specific binding of CTCF within the TAD only at the well-characterized ICR located 2-4 kb downstream of the H19 gene (Fig. 1a, arrow..).”
Line 121-123 “Within the 450 kb TAD that contains the Igf2-H19 domain, we particularly noticed the presence of 4 upstream clusters of bi-allelic CTCF binding, positioned between 50 – 250 kb away from the H19 ICR (Fig. 1a).”
I don’t understand how the authors are designating upstream and downstream. Throughout this section upstream and downstream are used without consideration of orientation H19 and Igf2 and the direction of transcription in the cluster. Traditionally, H19/Igf2 researched have considered 5’ of the start of transcription upstream and 3’ of transcription downstream. Thus, The ICR is upstream of H19 or 2-4kb 5’ of the transcription start, which is also between H19 and Igf2. The 4 biallelic clusters would be considered outside or downstream (or distal to) of the cluster. In fact, for this second case, I would argue using downstream or upstream is confusing. Please consider another means of designating these regions throughout the manuscript so that the reader doesn’t become confused. 

Line 293-294. Does differential DNA methylation of IG-DMR and Meg3DMR stay intact in differentiated site 2 deletion cells?

Lines 424-426: “In conclusion, our study reveals the importance of maternal-specific CTCF binding to structure a further layer of sub-TAD organization that is essential to override the inherent paternal organization and associated gene activation.” As mentioned in an earlier review, the TAD/sub-TAD structure associates with imprinted gene expression. There is no experiment in this paper that shows causation. Unlike their response in the response to reviews, the authors have not clarified this point within the text of the paper and are still ascribing imprinting function to the sub-TAD organization.

**Reviewer 2**

The authors addressed my questions thoughtfully.

**Reviewer 3**

All my comments were addressed

**Authors’ Point by Point response**

Remarks by Reviewer #1:

Remark 1: Line 95-97: "For the Igf2-H19 domain, we detected maternal allele-specific binding of CTCF within the TAD only at the well-characterized ICR located 2-4 kb downstream of the H19 gene (Fig. 1a, arrow..)."

Line 121-123 "Within the 450 kb TAD that contains the Igf2-H19 domain, we particularly noticed the presence of 4 upstream clusters of bi-allelic CTCF binding, positioned between 50 - 250 kb away from the H19 ICR (Fig. 1a)."

I don't understand how the authors are designating upstream and downstream. Throughout this section upstream and downstream are used without consideration of orientation H19 and Igf2 and the direction of transcription in the cluster. Traditionally, H19/Igf2 researched have considered 5' of the start of transcription upstream and 3' of transcription downstream. Thus, The ICR is upstream of H19 or 2-4kb 5' of the transcription start, which is also between H19 and Igf2. The 4 biallelic clusters would be considered outside or downstream (or distal to) of the cluster. In fact, for this second case, I would argue using downstream or upstream is confusing. Please consider another means of designating these regions throughout the manuscript so that the reader doesn't become confused.

*To accommodate the reviewer, we have replaced all mentions of upstream and downstream by centromeric and telomeric (including in the figures and the supplemental information). Due to the acrocentric nature of mouse chromosomes, these designations are unambiguous. Centromeric and telomeric orientations have been indicated in Figures 1 – 5 and S2 as well.*

Remark 2: Line 293-294. Does differential DNA methylation of IG-DMR and Meg3 DMR stay intact in differentiated site 2 deletion cells?

*To address this question, we have added two additional lines of evidence to Fig. S7, as discussed on page 13 of the manuscript:*

*- We determined DNA methylation levels of the IG-DMR and Meg3 DMR in our in-dept characterized clone 4 using methylation-sensitive endonuclease digestion (similar to Fig. S1C). These results confirm that DNA methylation at both loci is maintained at the expected 50% methylation level (Fig. S7D).*

*- The expression of Meg3 is directly dependent on the methylation status of the IG-DMR and Meg3 DMR. In the previous version of the manuscript, we already showed that Meg3 was expressed only from the maternal chromosome in all ESC and NPC lines with the CTCF site-2 deletion (Figs. S6B and S7C). To further support this observation, we have added representative single-molecule RNA FISH data to show that in clone 4 there is extensive expression of Meg3 in all the cells (Fig. S7D). Importantly, this finding implies that the Meg3 DMR-promoter region has remained active in all cells, and therefore should be unmethylated on the maternal allele. The paternal allele remains fully repressed in the NPCs.*

Remark 3: Lines 424-426: "In conclusion, our study reveals the importance of maternal-specific CTCF binding to structure a further layer of sub-TAD organization that is essential to override the inherent paternal organization and associated gene activation." As mentioned in an earlier review, the TAD/sub-TAD structure associates with imprinted gene expression. There is no experiment in this paper that shows causation. Unlike their response in the response to reviews, the authors have not clarified this point within the text of the paper and are still ascribing imprinting function to the sub-TAD organization.

*This sentence escaped our attention. We have replaced it by “In conclusion, our study reveals how maternal-specific CTCF binding structures a further layer of sub-TAD organization that overrides the inherent paternal 3D chromatin organization and thus directly coincides with the implementation of the maternal transcriptional programs.”*
